# Briaviolides K–N, New Briarane-Type Diterpenoids from Cultured Octocoral *Briareum violaceum*

**DOI:** 10.3390/md16030075

**Published:** 2018-02-27

**Authors:** Jing-Hao Xu, Kuei-Hung Lai, Yin-Di Su, Yu-Chia Chang, Bo-Rong Peng, Anders Backlund, Zhi-Hong Wen, Ping-Jyun Sung

**Affiliations:** 1 Graduate Institute of Marine Biology, National Dong Hwa University, Pingtung 94450, Taiwan; z0204478@gmail.com; 2 Planning & Research Division, National Museum of Marine Biology and Aquarium, Pingtung 94450, Taiwan; mos19880822@gmail.com (K.-H.L.); gobetter04@gmail.com (Y.-D.S.); jay0404@gmail.com (Y.-C.C.); pengpojung@gmail.com (B.-R.P.); 3 Division of Pharmacognosy, Department of Medicinal Chemistry, Uppsala University, 75123 Uppsala, Sweden; anders.backlund@fkog.uu.se; 4 Greenhouse Systems Technology Center, Central Region Campus, Industrial Technology Research Institute, Nantou 54041, Taiwan; 5 Department of Marine Biotechnology and Resources, National Sun Yat-sen University, Kaohsiung 80424, Taiwan; 6 Doctoral Degree Program in Marine Biotechnology, National Sun Yat-sen University, Kaohsiung 80424, Taiwan; 7 Doctoral Degree Program in Marine Biotechnology, Academia Sinica, Taipei 11529, Taiwan; 8 Chinese Medicine Research and Development Center, China Medical University Hospital, Taichung 40447, Taiwan; 9 Graduate Institute of Natural Products, Kaohsiung Medical University, Kaohsiung 80708, Taiwan

**Keywords:** *Briareum violaceum*, briarane, briaviolide, iNOS, COX-2, ChemGPS-NP

## Abstract

Four new briarane diterpenoids, briaviolides K–N (**1**–**4**), have been obtained from the cultured-type octocoral *Briareum violaceum*. Using a spectroscopic approach, the structures of briaranes **1**–**4** were identified. This study employed an in vitro model of lipopolysaccharide (LPS)-induced inflammation in the murine macrophage RAW 264.7 cell line, and found that among the four briaranes, briarane **2** possessed anti-inflammatory activity against inducible nitric oxide synthase (iNOS) and cyclooxygenase-2 (COX-2) protein expressions in cells. In addition, principal component analysis using the chemical global positioning system (ChemGPS) for natural products (ChemGPS-NP) was employed in order to analyze the structure-activity relationship (SAR), and the results indicated that the ring conformation of the compound has a leading role in suppressing the expressions of pro-inflammatory iNOS and COX-2 proteins in macrophages.

## 1. Introduction

**Figure 1:**
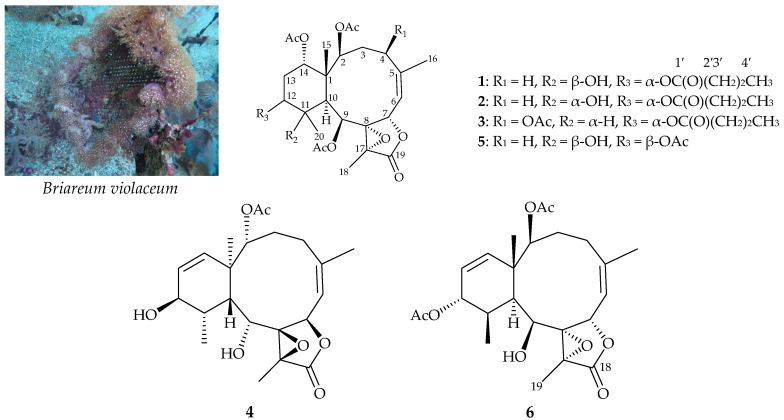
Cultured octocoral *B. violaceum* and structures of briaviolides K–N (**1**–**4**), excavatoid F (**5**), and (1S*,2S*,5*Z*,7S*,8S*,9S*,10S*,11R*,12R*,13*Z*,17R*)-2,12-diacetoxy-8,17-epoxy-9-hydroxybriara-5, 13-dien-18-one (**6**). The 1′2′3′4′ means the serial numbers of the carbon atom of the *n*-butyrate moiety.

The octocoral *Briareum violaceum* (Quoy & Gaimard, 1883) (family Briareidae) (Figure 1) [1] is a rich source of diterpenoids with a briarane carbon skeleton that often have complex structures and bioactivities [2,3,4,5,6]. In studies of the chemical constituents of cultured *B. violaceum*, four new briaranes, briaviolides K–N (**1**–**4**), were obtained (Figure 1). We isolated these compounds from cultured-type octocoral *B. violaceum*, determined their structures, and further studied their anti-inflammatory activities in terms of their inhibition of inducible oxide synthase (iNOS) and cyclooxygenase-2 (COX-2) in an in vitro pro-inflammatory macrophage culture model.

## 2. Results and Discussion

### 2.1. Chemical Identification of Isolated Briaranes

Briaviolide K (**1**) was obtained as an amorphous powder. The high-resolution electrospray ionization mass spectrum (HR-ESI-MS) of compound **1** showed a pseudomolecular peak at *m*/*z* 617.25684, which established the molecular formula C_30_H_42_O_12_ (calcd. for C_30_H_42_O_12_ + Na, 617.25685) indicating ten degrees of unsaturation. Absorption peaks at 3491, 1779, and 1732 cm^–1^ in the IR spectrum of **1** indicated a structure containing hydroxy, γ-lactone, and ester groups. Data from a distortionless enhancement of polarization transfer (DEPT) experiment and ^1^H NMR study, together with analysis of the molecular formula, suggested the existence of one exchangeable proton, which required the presence of one hydroxy group. A trisubstituted olefin was identified from two carbon signals (δ_C_ 146.2 and 117.4; C and CH, respectively), and a tetrasubstituted epoxide, containing a methyl substituent was confirmed from signals of two oxygenated quaternary carbons (δ_C_ 70.7 and 64.5; both C) and from the proton signal of a methyl (δ_H_ 1.70, 3H, s) in the ^13^C and ^1^H NMR spectrum of **1** (Table 1), respectively. In addition, five carbonyl resonances (δ_C_ 172.6, 170.7, 170.6, 170.4, and 169.4) further confirmed the existence of one γ-lactone and four other ester groups. On the other hand, the results of ^1^H NMR spectrum analysis indicated three acetate methyls (δ_H_ 2.22, 2.00, and 1.98; all 3H, s); the additional ester group was identified as an *n*-butyroxy group, and ^1^H–^1^H correlation spectroscopy (COSY) also revealed seven contiguous protons [δ_H_ 0.95 (3H, t), 1.65 (2H, sext), and 2.29 (2H, t); all *J* values 7.6 Hz]. Based on correlations identified by heteronuclear multiple bond coherence (HMBC) analysis, the carbon signal (δ_C_ 172.6, C) was found to be associated with the signal of the methylene protons (δ_H_ 2.29), and was consequently assigned as the carbon atom of the *n*-butyrate carbonyl. From the aforementioned data, metabolite **1** was identified as a compound with four rings.

^1^H–^1^H COSY spectrum analysis of **1** enabled determination of the proton sequences between H-2/H_2_-3/H_2_-4, H-6/H_3_-16 (by allylic coupling), H-6/H-7, and H-9/H-10. These results together with the HMBC correlations between H-2/C-1, C-4; H_2_-3/C-4; H_2_-4/C-5, C-6; H-7/C-5, C-6; H-9/C-1, C-7, C-8, C-10; and H-10/C-1, C-8, C-9, indicated a 10-membered ring connecting C-1 to C-10. At C-5, a vinyl methyl connected to this carbon was confirmed by HMBC correlations between H_3_-16/C-4, C-5, and C-6. In addition, a methylcyclohexane ring (attached at C-1 and C-10) linked to the 10-membered ring was elucidated by HMBC according to correlations between H-9/C-11; H-10/C-20; H-14/C-1; H_3_-20/C-10, C-11, C-12; and OH-11/C-10. The ring juncture methyl group at C-15 was located at C-1 based on the HMBC experiment, which showed correlations between H_3_-15/C-1, C-2, C-10, C-14; H-2/C-15; and H-10/C-15. At C-12, an *n*-butyrate ester was located according to connectivity between H-12 (δ_H_ 4.85) and the carbonyl carbon of the *n*-butyrate (δ_C_ 172.6). Furthermore, the other three acetoxy groups were located at C-2, C-9, and C-14, based on the HMBC correlations of the oxymethine protons at δ_H_ 5.17 (H-2), 5.74 (H-9), and 4.56 (H-14) with the ester carbonyls at δ_C_ 170.4, 169.4, and 170.6, respectively. The hydroxy proton signal occurring at δ_H_ 2.11 (1H, s) was found to be correlated in the HMBC spectrum with C-10, C-11, C-12, and C-20. Thus, we speculated that the hydroxy group was located at C-11. The aforementioned data, together with the HMBC correlations between H-7/C-19; H-9/C-17; and, H_3_-18/C-8, C-17, C-19, defined the molecular framework of **1**.

**Figure 2:**
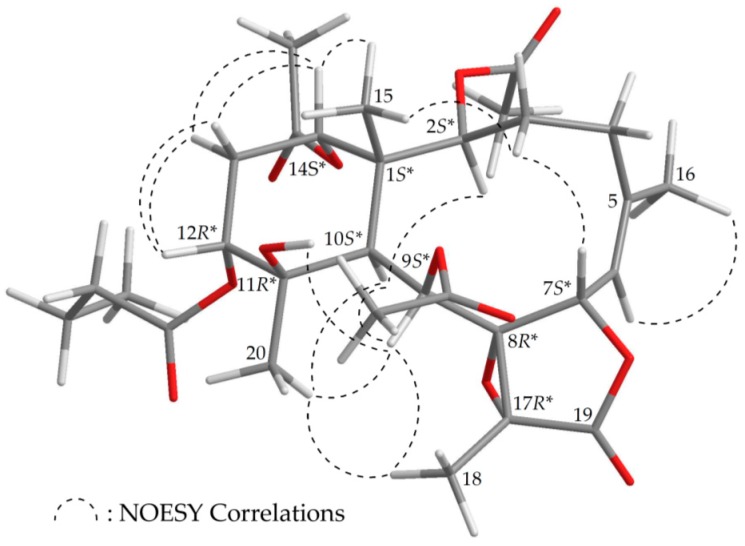
Model of **1** generated by computer-aided analyses based on MM2 force field calculations and selected protons with key NOESY correlations.

The stereochemistry of **1** was established by nuclear Overhauser effect spectroscopy (NOESY) analysis, which was further supported by MM2 force field analysis, demonstrating that the most stable conformation of **1** was as shown in Figure 2 [7]. H-2, H-9, H-10, and Me-20 protons were concluded to be located on the same face of the molecule, as these protons were correlated together, but were not correlated with H_3_-15 and H-12; as a result, they were assigned as α protons, as Me-15 and H-12 were β-substituents at C-1 and C-12, respectively. Due to the H-14 proton being correlated with H_3_-15, and not with H-10, this proton was of a β-orientation at C-14. Also, H_3_-18 was found to be associated with H-9 and H_3_-20, suggesting that the C-18 methyl in the γ-lactone moiety had a β-orientation. As H-9 was correlated with H-10, H_3_-18, H_3_-20, and OH-11, this also indicated that the 9-acetoxy group had a β-orientation. A correlation between a proton of the C-3 methylene (δ_H_ 2.62) and H_3_-15, but not H-2, suggested that the proton was a H-3β. A correlation between H-7 and H-3β, but not H-6, as well as a large coupling constant between H-7 and H-6 (*J* = 9.2 Hz), suggested that the dihedral angle between H-7 and H-6 was nearly 180°, and H-7 was β-oriented. The *Z*-configuration of the C-5/6 double bond was confirmed based on the fact that the C-6 olefin proton (δ_H_ 5.29) was correlated with the C-16 vinyl methyl (δ_H_ 2.05). Therefore, based on above findings, the relative configurations of the stereogenic centers of **1** were elucidated as 1S*,2S*,7S*,8R*, 9S*,10S*,11R*,12R*,14S*, and 17R* (Supplementary Materials, Figures S1–S9). It was found that the NMR signals of **1** were similar to those of a known briarane, excavatoid F (**5**) (Figure 1) [8], except that the signals that were corresponding to the β-acetoxy group at C-12 in **5** were replaced by signals for an α-*n*-butyroxy group in **1**. Thus, briaviolide K (**1**) was found to be the 12-deacetoxy-12-α-*n*-butyroxy derivative of **5**.

**Figure 3:**
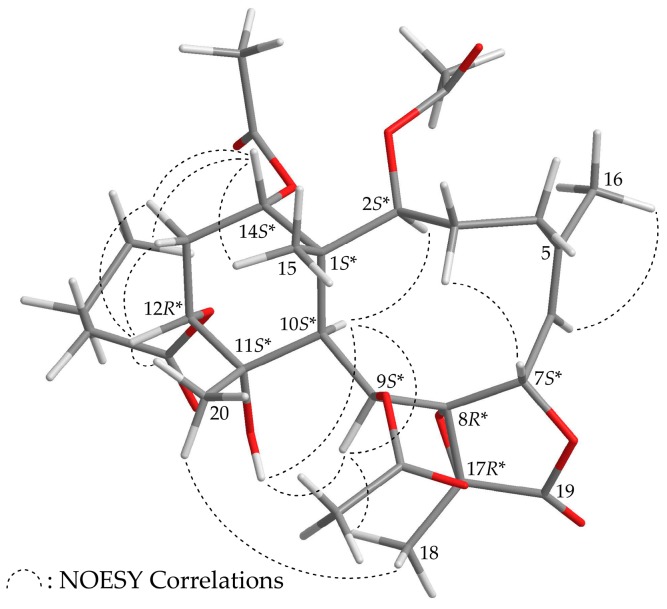
Model of **2** generated by computer-aided analyses based on MM2 force field calculations and selected protons with key NOESY correlations.

Briaviolide L (**2**) was found to have a molecular formula of C_30_H_42_O_12_ based on its HR-ESI-MS peak at *m/z* 617.25670 (calcd. for C_30_H_42_O_12_ + Na, 617.25685). The absorption peaks of the IR spectrum of **2** at 3374, 1779, and 1738 cm^–1^ were consistent with the presence of hydroxy, γ-lactone, and ester carbonyl groups. In addition, the analysis showed that the spectroscopic data of **1** and **2** were similar; however, comparison of the ^13^C NMR chemical shifts of the C-11 oxygenated quaternary carbon (δ_C_ 73.5) and Me-20 (δ_C_ 23.2) of **2** (Table 2) with those of **1** (δ_C_ 76.0, C-11; δ_C_ 30.0, Me-20) showed that the hydroxy group at C-11 in **2** was α-oriented. Therefore, this compound possessed the structure represented by formula **2**. Finally, based on structural data from a two-dimensional (2D) NMR experiment (Table 2) and a NOESY experiment (Supplementary Materials, Figures S10–S18), the stereogenic centers of **2** were assigned 1S*,2S*,7S*,8R*,9S*,10S*, 11S*,12R*,14S*, and 17R*, as shown in Figure 3.

The new diterpenoid, briaviolide M (**3**), was found to have a molecular formula of C_32_H_44_O_13_ based on its HR-ESI-MS peak at *m/z* 659.26733 (calcd. for C_32_H_44_O_13_ + Na, 659.26741). Its IR spectrum exhibited γ-lactone and ester carbonyls at 1782 and 1735 cm^–1^, respectively. Carbonyl resonances in the ^13^C NMR spectrum of **3** at δ_C_ 173.0, 170.5, 170.1, 170.0, 170.0, and 168.3 further confirmed that a γ-lactone and five other esters were present (Table 3). In the ^1^H NMR spectrum of **3** (Table 1), four acetate methyls were observed at δ_H_ 2.24, 2.05, 2.02, and 2.00 (each 3H × s). Acetoxy groups were positioned at C-2, C-4, C-9, and C-14 according to HMBC correlations between H-2 (δ_H_ 5.07), H-4 (δ_H_ 5.08), H-9 (δ_H_ 4.98), H-14 (δ_H_ 4.75), and the carbonyl carbons of the acetoxy groups at δ_C_ 170.1, 170.0, 168.3, 170.0, respectively. The additional ester was found to be an *n*-butyroxy group on the basis of ^1^H NMR studies and ^1^H–^1^H COSY analysis, which revealed seven contiguous protons (δ_H_ 0.96, 3H, t, *J* = 7.5 Hz; 1.64, 2H, sext, *J* = 7.5 Hz; 2.26, 2H, t, *J* = 7.5 Hz). Additionally, the carbon signal (at δ_C_ 173.0) was correlated with the signal (at δ_H_ 2.26) of the methylene protons in the HMBC spectrum, and therefore, it was consequently assigned as the carbon atom of the *n*-butyrate carbonyl. Finally, although no HMBC correlation was observed between H-12 (δ_H_ 4.83) and the *n*-butyrate carbonyl (δ_C_ 173.0), based on ^1^H–^1^H COSY correlations and characteristic NMR signals, the results suggested that the remaining *n*-butyroxy group should be positioned at C-12.

**Figure 4:**
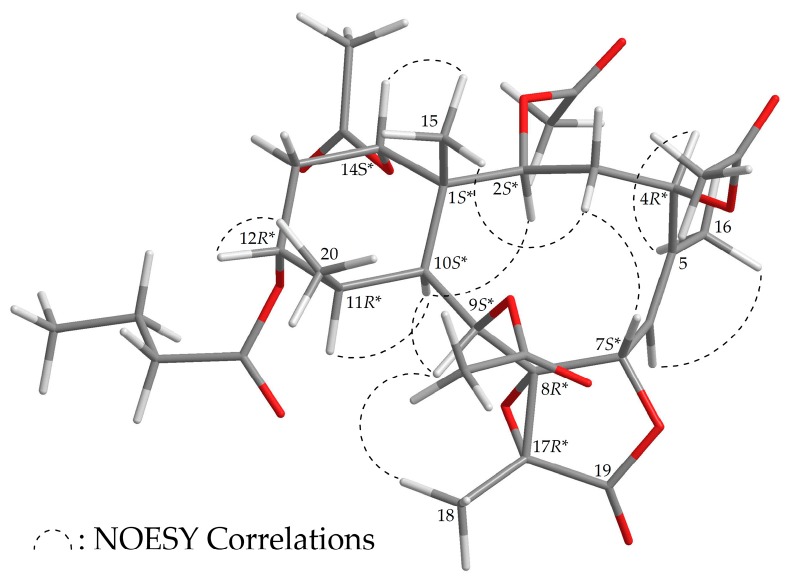
Model of **3** generated by computer-aided analyses based on MM2 force field calculations and selected protons with key NOESY correlations.

The stereochemistry of **3** was established based on the NOE interaction results from the NOESY experiment (Figure 4). In the NOESY spectrum of **3**, H-10 was associated with H-2, H-9, and H-11, while no associations were seen with Me-15 and Me-20, which suggested that these protons were positioned on the α face, due to the fact that the C-15 and C-20 methyls were β-substituents at C-1 and C-11, respectively. H-12 showed a correlation with Me-20, and H-14 was correlated with Me-15; there was no correlation between H-10/H-12 and H-10/H-14, demonstrating that the *n*-butyroxy and acetoxy groups at C-12 and C-14 were α-oriented. As one of the methylene protons at C-3 (δ_H_ 2.89) was correlated with H_3_-15, the proton was assigned as H-3β. Additionally, H-7 was assigned as β-oriented due to the existence of an association with H-3β. The *Z*- configuration of the C-5/6 double bond was established based on the interaction between H-6 and H_3_-16. Furthermore, correlation of H-4 with H_3_-16 indicated that the acetoxy group at C-4 was β-oriented, and H_3_-18 and H-9 correlation suggested that Me-18 should be positioned on the β-face in the γ-lactone ring (Supplementary Materials, Figures S19–S27). Thus, on the basis of the above results, **3** was found to possess the configuration 1S*,2S*,4R*,7S*,8R*,9S*,10S*,11R*,12R*,14S*, and 17R*.

Briaviolide N (**4**) was found to have a molecular formula of C_22_H_30_O_7_ based on its HR-ESI-MS peak at *m/z* 429.18819 (calcd. for C_22_H_30_O_7_ + Na, 429.18837). Its absorption peaks in the IR spectrum showed an ester carbonyl, a γ-lactone, and a broad OH stretch at 1731, 1779, and 3491 cm^–1^, respectively. Its ^13^C NMR spectrum indicated that a γ-lactone and an ester were present, as carbonyl resonances were observed δ_C_ 172.6 and 167.4, respectively (Table 4). The ^1^H NMR spectrum also indicated the presence of an acetate methyl (δ_H_ 2.23, 3H × s) (Table 4), and its NMR data were found to be similar to those of a known briarane analogue, (1S*,2S*,5*Z*,7S*,8S*,9S*,10S*,11R*,12R*,13*Z*,17R*)-2,12-diacetoxy-8,17-epoxy-9-hydroxybriara-5,13-dien-18-one (**6**) (Figure 1) [9,10], with the exception that the 12-acetoxy group in **6** was replaced by a hydroxy group in **4**. Based on HMBC correlations, an acetate group was concluded to be attached to C-2 in **4**, as an acetate carbonyl and an oxygen-bearing methine proton were observed at δ_C_ 167.4 and δ_H_ 4.83, respectively (Table 4). The locations of the functional groups were further confirmed by other HMBC correlations (Supplementary Materials, Figures S29–S36). Additionally, the stereogenic centers of **4** were found to be the same as those of **6**, as the ^1^H and ^13^C NMR chemical shifts, proton-proton coupling data, and NOESY correlations of **4** and **6** matched. However, the optical rotation value of **4** ([α]${}_{D}^{25}$ +22 (*c* 0.01, CHCl_3_)) was substantially different from that of **6** ([α]${}_{D}$ –44.9 (*c* 0.31) [9]; [α]${}_{D}^{25}$ –42 (*c* 0.3, CHCl_3_) [10]), indicating that **4** was found to be the enantiomer of the 12-deacetoxy-12-hydroxy derivative of **6**.

### 2.2. Anti-Inflammatory Activities of the Isolated Briaranes

**Figure 5:**
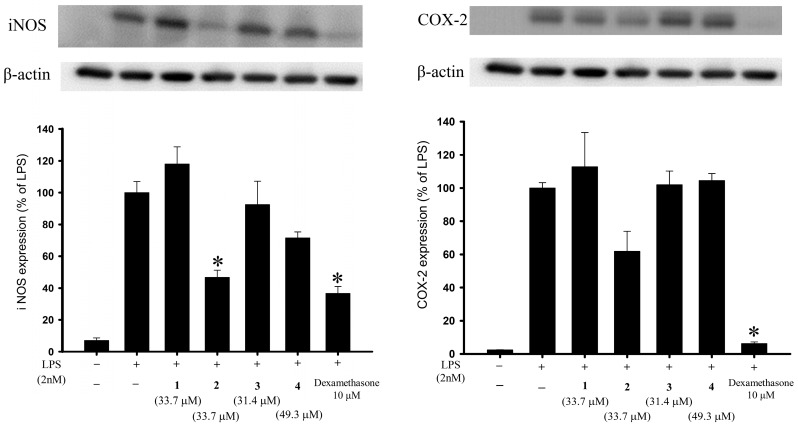
Effects of compounds **1**–**4** on the expression of pro-inflammatory iNOS and COX-2 proteins in murine RAW264.7 macrophage cell. Using immunoblot analysis, briarane **2** was demonstrated to reduce lipopolysaccharide (LPS)-induced expressions of these two pro- inflammatory proteins. Data were normalized to those of cells treated with LPS alone, and cells treated with dexamethasone (10 μM) were used as a positive control (which has been shown to reduce the levels of iNOS and COX-2 to 36.52% and 6.18%, respectively). Data are expressed as the mean ± SEM (*n* = 3). * Significantly different from cells treated with LPS (*p* < 0.05).

Anti-inflammatory activity assays using an in vitro cell culture model were performed in this study, and western blot analysis was employed to evaluate the changes in pro-inflammatory iNOS and COX-2 proteins in a lipopolysaccharide (LPS)-stimulated pro-inflammatory response in a murine macrophage RAW264.7 cell line. As compared with cells stimulated with LPS alone, the macrophages treated with a concentration of 20 μg/mL, briarane **2** exhibited reduced levels of iNOS and COX-2 to 46.68% and 61.81%, respectively (Figure 5 and Table 5). Using trypan blue staining to measure the cytotoxic effects of the compounds, it was observed that briaviolides K–N (**1**–**4**) did not induce significant cytotoxicity in RAW264.7 macrophage cells. Briarane **2** showed anti- inflammatory activities against the expressions of these two pro-inflammatory proteins. Briarane **1** was found to be inactive in terms of reducing the expressions of the above two pro-inflammatory proteins, indicating that the anti-inflammatory activity of these compounds is largely dependent on the stereochemistry of the hydroxy group at C-11.

### 2.3. ChemGPS-NP-Based Analysis of the Active Briaranes

ChemGPS-NP, a PCA (Principle Component Analysis)-based model applied to natural products, was first developed by the Backlund group in 2007 [11], and an entire online analysis system that provides the eight principal components (PCs) was further established by the same group in 2009 [12]. In the last few years, this computational tool has been employed to analyze structure-activity relationships (SAR) [13,14] and to further investigate promising pharmacological targets, guiding natural product drug discovery [15,16,17].

**Figure 6:**
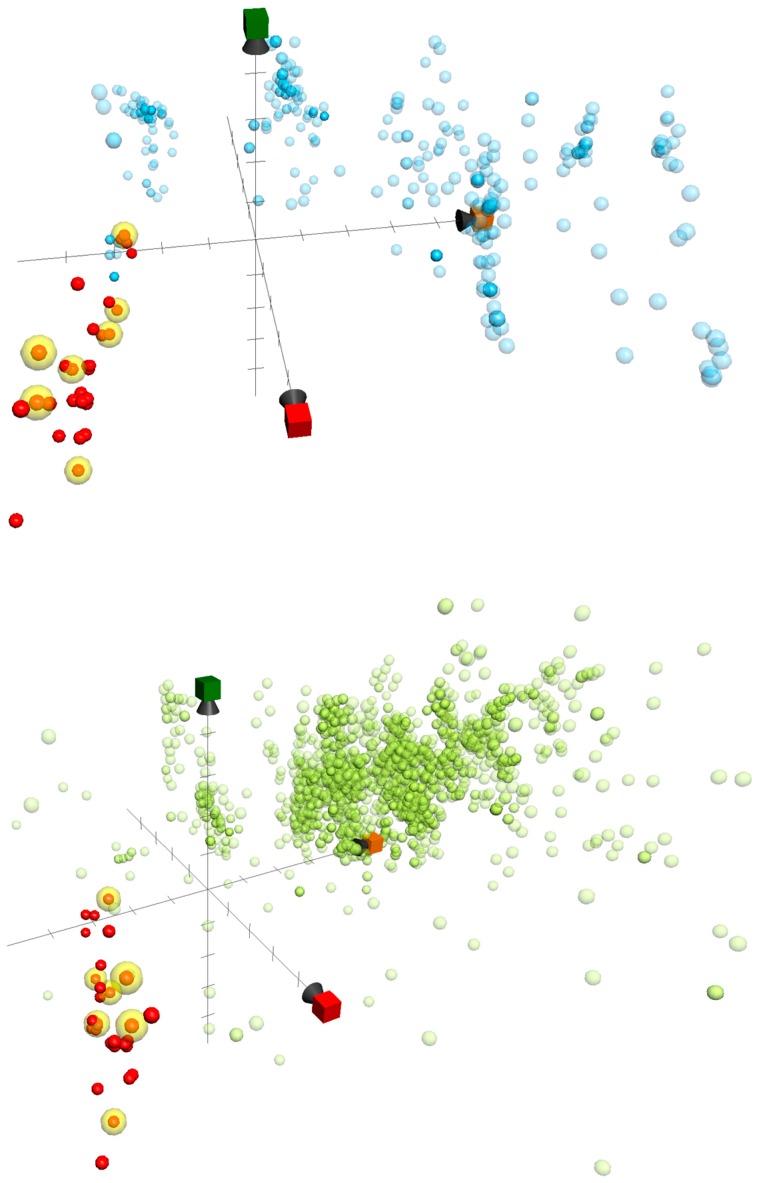
ChemGPS-NP-based analysis of the chemical space plotted for a series of briarane-type diterpenoids reported by our lab (red and yellow, which were active in inhibition assays of iNOS and COX-2), as well as previously studied 342 iNOS (light blue) and 2,592 COX-2 (light green) inhibitors sorted from the ChEMBL database. In the score plot of the three dimensions (principle component), PC1 (red) describes the size, shape, and polarizability; PC2 (orange) represents the aromatic- and conjugation-related properties; and PC3 (green) depicts lipophilicity, polarity, and H-bond capacity.

Herein, we used ChemGPS-NP to determine the anti-inflammatory activity-related chemical relationships between the briaranes reported in the current study and those that were reported in previous publications of our group [18,19,20,21,22,23,24,25]. In total, 32 briarane-type diterpenoids, including briarenolides H–Y [18,19,20,21,22], ZI–ZVI [23], briarenols B–E [24,25], and briaviolides K–N (**1**–**4**), were mapped into the chemical space based on PC score predictions of the online tool ChemGPS-NP_Web_ [12] (http:// chemgps.bmc.uu.se) (Figure 6). Moreover, previously disclosed inhibitors targeting inflammation-related proteins iNOS and COX-2 were sorted out from the Chemistry database of European Molecular Biology Laboratory (ChEMBL) online database (https://www. ebi.ac.uk/ chembl/), and as well populated on the analyzing graphic. The active compounds were distributed irregularly in the briarane cluster, and were also located incoherently to either the iNOS or COX-2 inhibitor group. As compound **2** exhibited entirely opposite activity when compared to **1**, with the only difference being the configuration of 11-hydroxy group, we surmised that the inhibitory effects of the compounds on these two pro-inflammatory proteins might be affected by the conformation of the briarane rings, and the configuration of attached groups many affect the compound’s bioactivity. Additionally, the results also indicated a completely different group of compounds in the ChemGPS-NP chemical space that exhibited potent anti-inflammatory properties. This bioactive relevant pattern in the chemical space could be applied in the future navigation of anti-inflammatory lead discovery using ChemGPS-NP.

## 3. Experimental Section

### 3.1. General Experimental Procedures

NMR spectra were recorded on a Jeol Resonance ECZ400S (Tokyo, Japan) or a Varian Inova 500 (Palo Alto, CA, USA) NMR spectrometers using the residual CHCl_3_ signal (δ_H_ 7.26 ppm) as an international standard for ^1^H NMR and CDCl_3_ (δ_C_ 77.1 ppm) for ^13^C NMR, respectively. Coupling constants (*J*) are presented in Hz. Column chromatography, IR spectra, ESI-MS and HR-ESI-MS, melting point, and optical rotation properties were performed as described in our previous study [18,19,20,21,22,24]. Column chromatograph was performed on silica gel (230–400 mesh, Merck, Darmstadt, Germany). Thin layer chromatograph (TLC) was carried out on precoated Kieselgel 60 F_254_ (0.25 mm, Merck); spots were visualized by spraying with 10% H_2_SO_4_ solution followed by heating. Normal-phase HPLC (NP-HPLC) was performed using a system comprised of a Hitachi L-7100 pump (Hitachi Ltd., Tokyo, Japan) and a Rheodyne 7725 injection port (Rheodyne LLC, Rohnert Park, CA, USA). A semi-preparative normal-phase column (Supelco Ascentis Si Cat#:581514-U, 25 cm × 10 mm, 5 μM, Sigma-Aldrich, St. Louis, MO, USA) was used for NP-HPLC. Reverse-phase HPLC (RP-HPLC) was performed using a system equipped with a Hitachi L-2130 pump, a Hitachi L-2455 photodiode array detector, a Rheodyne 7725 injection port, and a 250 mm × 21.2 mm column (5 μM, Luna RP-18e; Phenomenex Inc., Torrance, CA, USA) or a 25 cm × 10 mm column (5 μM; Ascentis C18 Cat#:581343-U, Sigma-Aldrich).

### 3.2. Animal Material

Specimens of the octocoral *B. violaceum* were collected from the waters off Taiwan, and relocated to a 270-ton cultivation tank located in the National Museum of Marine Biology and Aquarium (NMMBA), Taiwan, in 2011; the material used for this study was of the cultured-type *B. violaceum,* collected from the tank in December 2016. This organism was identified by comparison with previous descriptions [1]. A voucher specimen was deposited in the NMMBA, Taiwan (NMMBA-CSC-002).

### 3.3. Extraction and Isolation

Sliced bodies of *B. violaceum* (wet and dry weights, 251 and 95.0 g, respectively) were extracted with a mixture of organic solven (MeOH:CH_2_Cl_2_ = 1:1; volume ratio). The resulting 9.79 g extract was partitioned between ethyl acetate (EtOAc) and H_2_O. The EtOAc layer (3.30 g) was separated on silica gel and eluted with a mixture of *n*-hexane/EtOAc (stepwise from 100:1 to 100% EtOAc; volume ratio) to yield fifteen subfractions A–O. Fraction L was separated by silica gel column chromatography and then eluted with a mixture of *n*-hexane/acetone (stepwise 8:1 to 100% acetone; volume ratio) to afford twelve subfractions L1–L12. Fraction L4 was separated by silica gel column chromatography and then eluted with CH_2_Cl_2_/acetone (stepwise 80:1 to 100% acetone; volume ratio) to afford fourteen subfractions L4A–L4N. Fraction L4G was purified by NP-HPLC using a mixture of CH_2_Cl_2_/acetone (*v*:*v* = 30:1 of volume ratio at a flow rate of 2.0 mL/min) to afford **4** (0.2 mg) and five subfractions L4G1–L4G5. Fraction L4G2 was purified by RP-HPLC using a mixture of MeOH/ H_2_O (*v*:*v* = 80:20 of volume ratio at a flow rate of 1.0 mL/min) to yield **2** (0.7 mg) and **3** (0.8 mg). Fraction L4J was purified by RP-HPLC using a mixture of MeOH/H_2_O (*v*:*v* = 75:25 of volume ratio at a flow rate of 5.0 mL/min) to yield **1** (0.7 mg).

Briaviolide K (**1**): amorphous powder; mp 78–81 °C; [α]${}_{D}^{25}$ −18 (*c* 0.05, CHCl_3_); IR (neat) ν_max_ 3491, 1779, 1732 cm^−1^; ^1^H (400 MHz, CDCl_3_) and ^13^C (100 MHz, CDCl_3_) NMR data (see Table 1); ESI-MS: *m*/*z* 617 [M + Na]^+^; HR-ESI-MS: *m*/*z* 617.25684 (calcd. for C_30_H_42_O_12_ + Na, 617.25685).

Briaviolide L (**2**): amorphous powder; mp 78–81 °C; [α]${}_{D}^{25}$ −17 (*c* 0.04, CHCl_3_); IR (neat) ν_max_ 3374, 1779, 1738 cm^−1^; ^1^H (400 MHz, CDCl_3_) and ^13^C (100 MHz, CDCl_3_) NMR data (see Table 2); ESI-MS: *m*/*z* 617 [M + Na]^+^; HR-ESI-MS: *m*/*z* 617.25670 (calcd. for C_30_H_42_O_12_ + Na, 617.25685).

Briaviolide M (**3**): amorphous powder; mp 121–124 °C; [α]${}_{D}^{25}$ −6 (*c* 0.03, CHCl_3_); IR (neat) ν_max_ 1782, 1735 cm^−1^; ^1^H (500 MHz, CDCl_3_) and ^13^C (125 MHz, CDCl_3_) NMR data (see Table 3); ESI-MS: *m*/*z* 659 [M + Na]^+^; HR-ESI-MS: *m*/*z* 659.26733 (calcd. for C_32_H_44_O_13_ + Na, 659.26741).

Briaviolide N (**4**): amorphous powder; mp 90–93 °C; [α]${}_{D}^{25}$ +22 (*c* 0.01, CHCl_3_); IR (neat) ν_max_ 3491, 1779, 1731 cm^−1^; ^1^H (400 MHz, CDCl_3_) and ^13^C (100 MHz, CDCl_3_) NMR data (see Table 4); ESI-MS: *m*/*z* 429 [M + Na]^+^; HR-ESI-MS: *m*/*z* 429.18819 (calcd. for C_22_H_30_O_7_ + Na, 429.18837).

### 3.4. Molecular Mechanics Calculations

The implementation of the MM2 force field [7] in ChemBio 3D Ultra software from Cambridge Soft Corporation (ver. 12.0, Cambridge, MA, USA) was used to create the molecular models.

### 3.5. In Vitro Anti-Inflammatory Assay

Murine macrophage-like RAW264.7 cell line was purchased from the American Type Culture Collection (ATCC, No TIB-71) (Manassas, VA, USA). The in vitro model of the assay, which was used to evaluate the anti-inflammatory activities of compounds **1**–**4**, was performed using RAW264.7 cells pre-treated with compounds **1**–**4** then incubated with LPS to induce pro- inflammatory protein expressions. The inhibition effects of the compounds on the expressions of LPS-induced pro-inflammatory iNOS and COX-2 proteins in the cells were assessed using western blot analysis [26,27,28]. Briefly, RAW264.7 cells were untreated or pre-treated with 20 μg/mL compounds **1**–**4** (33.7, 33.7, 31.4, and 49.3 μM, respectively) or 10 μM dexamethasone as a positive control for 10 min, followed by adding 10 ng/mL LPS (2 nM), and incubating for 16 hr. The cell lysates were then collected for western blotting analysis. Protein expression levels were calculated based on the intensities of the bands on the blots representing the immunoreactivity to the iNOS and COX-2 antibodies. The cytotoxic effects of compounds **1**–**4** were also evaluated using a trypan blue exclusion assay [27,28]. Statistical analyses were performed using one-way analysis of variance (ANOVA), and data were further processed by the Student–Newman–Keuls *post hoc* test for multiple comparisons. A *p*-value of <0.05 was considered to indicate a significant difference between two treatments.

### 3.6. ChemGPS-NP Analysis

ChemGPS-NP (http://chemgps.bmc.uu.se), a tool for navigation in biologically-relevant chemical space, was employed in this study. The tool contains eight principal components (PCs) that consider data from 35 chemical descriptors describing physical-chemical properties. The data were obtained from carefully-selected information covering all of the important aspects, including flexibility, hydrogen-bond capacity, lipophilicity, polarity, polarizability, rigidity, size, and shape. Using this tool, ChemGPS-NP prediction scores were determined for the briarane-type diterpenoids from the online tool ChemGPS-NP_Web_ [12] (http://chemgps.bmc.uu.se). The method was based on their structural information as a simplified molecular input line entry specification (SMILES) derived via ChemBioDraw (ver. #16.0, Cambridge Software, St. Neots, UK). Grapher (ver. 2.6, Mac OS, Cupertino, CA, USA) was used to plot the compounds into the ChemGPS-NP chemical property space, together with previously reported information regarding iNOS and COX-2 inhibitors from the ChEMBL database. The selecting active agents was sorted out based on selecting IC_50_ < 1000 nM as the threshold from data obtained from ChEMBL.

## 4. Conclusions

The octocoral *B. violaceum* has been reported to demonstrate a wide structural diversity of interesting briarane-type diterpenoids with extensive pharmacological properties [29]. Our continuous search for more potent anti-inflammatory briaranes resulted in an acquisition of four new metabolites, named briaviolides K–N (**1**–**4**). Their anti-inflammatory capabilities were further examined by targeting to two pro-inflammatory proteins, iNOS or COX-2. Among all of the tested compounds, briaviolide L (**2**) exhibited the most potent effect in inhibiting iNOS production. Noteworthily, compounds **1** and **2**, with the only difference of the 11-hydroxy configuration, were found to exhibit entirely contrary effects in anti-inflammatory evaluation. The consecutive ChemGPS-NP analysis on a series of briaranes also supported the presumption that the anti-inflammatory activity of these briaranes may be affected by their ring conformations. In addition, the cultured octocoral *Briareum violaceum* maintained in the National Museum of Marine Biology and Aquarium provided a sustainable furnish to execute the first steps of future drug discovery.

## Figures and Tables

**Table 1:** Data of ^1^H (400 MHz, CDCl_3_) and ^13^C (100 MHz, CDCl_3_) NMR and ^1^H–^1^H COSY and HMBC correlations for briaviolide K (**1**).

Position	δ_H_ (*J* in Hz)	δ_C_, Multiple	^1^H–^1^H COSY	HMBC
1		46.5, C		
2	5.17 d (8.0)	75.4, CH	H_2_-3	C-1, C-4, C-15, acetate carbonyl
3α/β	1.73 m; 2.62 ddd (15.2, 15,2, 6.0)	32.3, CH_2_	H-2, H_2_-4	C-4
4/4′	1.95 m; 2.53 br d (15.2)	28.7, CH_2_	H_2_-3	C-5, C-6
5		146.2, C		
6	5.29 d (9.2)	117.4, CH	H-7, H_3_-16	n. o. ^a^
7	5.37 d (9.2)	75.1, CH	H-6	C-5, C-6, C-19
8		70.7, C		
9	5.74 d (2.0)	68.0, CH	H-10	C-1, C-7, C-8, C-10, C-11, C-17,
				acetate carbonyl
10	2.34 br s	44.9, CH	H-9	C-1, C-8, C-9, C-15, C-20
11		76.0, C		
12	4.85 dd (3.6, 2.4)	75.0, CH	H_2_-13	*n*-butyrate carbonyl
13/13′	2.01 m; 2.19 m	24.3, CH_2_	H-12, H-14	C-12
14	4.56 dd (2.8, 2.8)	74.9, CH	H_2_-13	C-1, acetate carbonyl
15	1.28 s	14.8, CH_3_		C-1, C-2, C-10, C-14
16	2.05 br s	27.0, CH_3_	H-6	C-4, C-5, C-6
17		64.5, C		
18	1.70 s	10.2, CH_3_		C-8, C-17, C-19
19		170.7, C		
20	1.29 s	30.0, CH_3_		C-10, C-11, C-12
OAc-2		170.4, C		
	1.98 s	21.5, CH_3_		Acetate carbonyl
OAc-9		169.4, C		
	2.22 s	21.2, CH_3_		Acetate carbonyl
OAc-14		170.6, C		
	2.00 s	21.5, CH_3_		Acetate carbonyl
*n*-OC(O)Pr-12		172.6, C		
	2.29 t (7.6)	36.4, CH_2_	H_2_-3′	C-1′, C-3′, C-4′
	1.65 sext (7.6)	18.3, CH_2_	H_2_-2′, H_3_-4′	C-1′, C-2′, C-4′
	0.95 t (7.6)	13.7, CH_3_	H_2_-3′	C-2′, C-3′
OH-11	2.11 s			C-10, C-11, C-12, C-20

^a^ n. o. = not observed.

**Table 2:** Data of ^1^H (400 MHz, CDCl_3_) and ^13^C (100 MHz, CDCl_3_) NMR and ^1^H–^1^H COSY and HMBC correlations for briaviolide L (**2**).

Position	δ_H_ (*J* in Hz)	δ_C_, Multiple	^1^H–^1^H COSY	HMBC
1		47.3, C		
2	5.23 d (8.4)	75.0, CH	H_2_-3	C-1, C-4, C-14, C-15, acetate carbonyl
3α/β	1.69 m; 2.64 ddd (15.2, 15.2, 5.6)	31.4, CH_2_	H-2, H_2_-4	C-2
4/4′	1.93 m; 2.49 br d (15.2)	28.4, CH_2_	H_2_-3	C-5
5		144.3, C		
6	5.20 d (8.0)	118.6, CH	H-7, H_3_-16	C-5, C-8
7	5.19 d (8.0)	75.0, CH	H-6	C-6, C-8
8		70.7, C		
9	5.83 s	67.4, CH	H-10	C-1, C-7, C-8, C-10, C-11, C-17, acetate carbonyl
10	2.41 s	45.5, CH	H-9	C-1, C-2, C-8, C-12, C-14, C-15, C-20
11		73.5, C		
12	4.80 dd (2.8, 2.4)	73.8, CH	H_2_-13	C-10, C-11, C-14, C-20, *n*-butyrate carbonyl
13/13′	1.96 m; 2.26 m	25.8, CH_2_	H-12, H-14	C-11, C-12, C-14
14	4.68 dd (2.8, 2.8)	74.2, CH	H_2_-13	C-1, C-10, C-15, acetate carbonyl
15	1.22 s	14.3, CH_3_		C-1, C-2, C-10, C-14
16	1.99 s	27.2, CH_3_	H-6	C-4, C-5, C-6
17		66.3, C		
18	1.77 s	10.4, CH_3_		C-8, C-17, C-19
19		170.4, C		
20	1.22 s	23.2, CH_3_		C-10, C-11, C-12
OAc-2		170.1, C		
	1.98 s	21.4, CH_3_		Acetate carbonyl
OAc-9		168.2, C		
	2.22 s	21.2, CH_3_		Acetate carbonyl
OAc-14		170.1, C		
	1.99 s	21.5, CH_3_		Acetate carbonyl
*n*-OC(O)Pr-12		172.5, C		
	2.33 t (7.2)	36.3, CH_2_	H_2_-3′	C-1′, C-3′, C-4′
	1.67 sext (7.2)	18.3, CH_2_	H_2_-2′, H_3_-4′	C-1′, C-2′, C-4′
	0.97 t (7.2)	13.7, CH_3_	H_2_-3′	C-2′, C-3′
OH-11	1.96 s			C-11, C-12, C-20

**Table 3:** Data of ^1^H (500 MHz, CDCl_3_) and ^13^C (125 MHz, CDCl_3_) NMR and ^1^H–^1^H COSY and HMBC correlations for briaviolide M (**3**).

Position	δ_H_ (*J* in Hz)	δ_C_, Multiple	^1^H–^1^H COSY	HMBC
1		46.1, C		
2	5.07 d (7.5)	73.0, CH	H_2_-3	C-1, C-3, C-4, C-15,
				acetate carbonyl
3α/β	2.02 (15.0, 7.5, 5.5); 2.89 dd (15.0, 13.0)	37.7, CH_2_	H-2, H-4	C-2, C-4, C-5
4	5.08 dd (13.0, 5.5)	73.0, CH	H_2_-3	C-5, C-6, acetate carbonyl
5		143.7, C		
6	5.40 d (9.0)	123.1, CH	H-7	C-4
7	5.57 d (9.0)	73.7, CH	H-6	C-6
8		70.3, C		
9	4.98 br s	72.5, CH	n. o. ^b^	C-1, C-7, C-8, C-11, C-17,
				acetate carbonyl
10	2.64 dd (5.0, 1.5)	37.3, CH	H-11	C-1, C-8, C-11
11	2.04 m	42.0, CH	H-10, H-12, H_3_-20	n. o.
12	4.83 ddd (3.5, 3.0, 3.0)	71.4, CH	H-11, H_2_-13	n. o.
13α/β	1.94 br d (16.0); 2.09 ddd (16.0, 3.0, 3.0)	25.4, CH_2_	H-12, H-14	n. o.
14	4.75 dd (3.0, 3.0)	73.9, CH	H_2_-13	Acetate carbonyl
15	1.23 s	15.4, CH_3_		C-1, C-10, C-14
16	2.17 s	25.4, CH_3_		C-4, C-5, C-6
17		64.5, C		
18	1.62 s	10.8, CH_3_		C-8, C-17, C-19
19		170.5, C		
20	1.11 d (7.5)	15.0, CH_3_		C-10, C-11, C-12
OAc-2		170.1, C ^a^		
	2.05 s ^a^	21.1, CH_3_		Acetate carbonyl
OAc-4		170.0, C ^a^		
	2.02 s ^a^	21.1, CH_3_		Acetate carbonyl
OAc-9		168.3, C		
	2.24 s	21.4, CH_3_		Acetate carbonyl
OAc-14		170.0, C ^a^		
	2.00 s ^a^	21.1, CH_3_		Acetate carbonyl
*n*-OC(O)Pr-12		173.0, C		
	2.26 t (7.5)	36.5, CH_2_	H_2_-3′	C-1′, C-3′ C-4′
	1.64 sext (7.5)	18.5, CH_2_	H_2_-2′, H_3_-4′	C-1′, C-2′ C-4′
	0.96 t (7.5)	13.7, CH_3_	H_2_-3′	C-2′, C-3′

^a^ data exchangeable; ^b^ n. o. = not observed.

**Table 4:** Data of ^1^H (400 MHz, CDCl_3_) and ^13^C (100 MHz, CDCl_3_) NMR and ^1^H–^1^H COSY and HMBC correlations for briaviolide N (**4**).

Position	δ_H_ (*J* in Hz)	δ_C_, Multiple	^1^H–^1^H COSY	HMBC
1		43.3, C		
2	4.83 d (8.4)	82.3, CH	H_2_-3	C-4, C-10, C-14, acetate carbonyl
3/3′	2.27 m, 1.87 m	22.7, CH_2_	H-2, H_2_-4	C-1, C-2
4/4′	1.92 m, 2.54 dd (14.8, 4.4)	24.7, CH_2_	H_2_-3	C-2, C-5
5		143.1, C		
6	5.34 d (8.8)	121.0, CH	H-7	C-4
7	5.65 d (8.8)	73.7, CH	H-6	C-5
8		70.9, C		
9	3.73 dd (10.0, 6.0)	68.5, CH	H-10, OH-9	C-8, C-11
10	3.49 dd (10.0, 3.6)	34.6, CH	H-9, H-11	n. o. ^a^
11	2.16 m	35.7, CH	H-10, H-12, H_3_-20	n. o.
12	3.92 dd (6.0, 1.6)	68.2, CH	H-11, H-13	n. o.
13	5.90 dd (10.0, 6.0)	126.5, CH	H-12, H-14	n. o.
14	5.37 d (10.0)	138.1, CH	H-13	C-10, C-12
15	1.20 s	19.2, CH_3_		C-1, C-2, C-10, C-14
16	1.78 s	23.4, CH_3_		C-4, C-5, C-6
17		58.5, C		
18	1.60 s	9.4, CH_3_		C-8, C-17, C-19
19		172.6, C		
20	0.90 d (7.2)	13.4, CH_3_	H-11	C-10, C-11, C-12
OAc-2		167.4, C		
	2.23 s	20.8, CH_3_		Acetate carbonyl
OH-9	4.92 d (6.0)		H-9	C-8, C-10

^a^ n. o. = not observed.

**Table 5:** Effects of briaranes **1**–**4** on LPS-induced iNOS and COX-2 protein expressions in macrophages.

Compound	iNOS	COX-2
	Expression (% of LPS Group)	Expression (% of LPS Group)
Control	7.08 ± 1.55	2.41 ± 0.13
LPS	100 ± 6.96	100 ± 3.26
1	118.02 ± 10.81	112.77 ± 20.69
2	46.68 ± 4.56	61.81 ± 12.14
3	92.49 ± 14.67	101.95 ± 8.22
4	71.49 ± 3.78	104.51 ± 4.22
DEX ^a^	36.52 ± 4.53	6.18 ± 1.05

^a^ Dexamethasone (DEX, 10 μM) was used as a positive control.

## Supplementary Materials

The following are available online at http://www.mdpi.com/1660-3397/16/3/75/s1, HR-ESI-MS, IR, 1D (^1^H NMR, ^13^C NMR, and DEPT spectra), and 2D (HSQC, ^1^H−^1^H COSY, HMBC, and NOESY spectra) NMR spectra of new compounds **1**–**4**.
