# Supplementary material for: Briaviolides K–N, New Briarane-Type Diterpenoids from Cultured Octocoral Briareum violaceum
Source: Mar Drugs. 2018 Feb 27;16(3):75. doi: 10.3390/md16030075 (PMC5867619; doi:10.3390/md16030075)
Supplement: Supplementary file 1 [file marinedrugs-16-00075-s001.pdf]

Supporting Information

Briaviolides K–N, New Briarane-type Diterpenoids from Cultured Octocoral *Briareum violaceum*

Jing-Hao Xu [1,2], Kuei-Hung Lai [2,3], Yin-Di Su [2,4], Yu-Chia Chang [2,4,5], Bo-Rong Peng [2,6,7], Anders Backlund [3], Zhi-Hong Wen [5,*], and Ping-Jyun Sung [1,2,5,8,9,*]

1 Graduate Institute of Marine Biology, National Dong Hwa University, Pingtung 94450, Taiwan
2 Planning & Research Division, National Museum of Marine Biology and Aquarium, Pingtung 94450, Taiwan
3 Division of Pharmacognosy, Department of Medicinal Chemistry, Uppsala University, Uppsala 75123, Sweden
4 Greenhouse Systems Technology Center, Central Region Campus, Industrial Technology Research Institute, Nantou 54041, Taiwan
5 Department of Marine Biotechnology and Resources, National Sun Yat-sen University, Kaohsiung 80424, Taiwan;
6 Doctoral Degree Program in Marine Biotechnology, National Sun Yat-sen University, Kaohsiung 80424, Taiwan; pengpojung@gmail.com
7 Doctoral Degree Program in Marine Biotechnology, Academia Sinica, Taipei 11529, Taiwan
8 Graduate Institute of Natural Products, Kaohsiung Medical University, Kaohsiung 80708, Taiwan
9 Chinese Medicine Research and Development Center, China Medical University Hospital, Taichung 40447, Taiwan

No	Content	page
S1.	HR-ESI-MS spectrum of compound **1**.	3
S2.	IR spectrum of compound **1**.	3
S3.	^{1}H NMR spectrum (400 MHz) of compound **1** in $CDCl_3$.	4
S4.	^{13}C NMR spectrum (100 MHz) of compound **1** in $CDCl_3$.	4
S5.	DEPT spectrum of compound **1** in $CDCl_3$.	5
S6.	gHSQC spectrum of compound **1** in $CDCl_3$.	5
S7.	^{1}H–^{1}H gCOSY spectrum of compound **1** in $CDCl_3$.	6
S8.	gHMBC spectrum of compound **1** in $CDCl_3$.	6
S9.	NOESY spectrum of compound **1** in $CDCl_3$.	7
S10.	HR-ESI-MS spectrum of compound **2**.	7
S11.	IR spectrum of compound **2**.	8
S12.	^{1}H NMR spectrum (400 MHz) of compound **2** in $CDCl_3$.	8
S13.	^{13}C NMR spectrum (100 MHz) of compound **2** in $CDCl_3$.	9
S14.	DEPT spectrum of compound **2** in $CDCl_3$.	9
S15.	gHSQC spectrum of compound **2** in $CDCl_3$.	10
S16.	^{1}H–^{1}H gCOSY spectrum of compound **2** in $CDCl_3$.	10
S17.	gHMBC spectrum of compound **2** in $CDCl_3$.	11
S18.	NOESY spectrum of compound **2** in $CDCl_3$.	11
S19.	HR-ESI-MS spectrum of compound **3**.	12
S20.	IR spectrum of compound **3**.	12
S21.	^{1}H NMR spectrum (500 MHz) of compound **3** in $CDCl_3$.	13
S22.	^{13}C NMR spectrum (125 MHz) of compound **3** in $CDCl_3$.	13
S23.	DEPT spectrum of compound **3** in $CDCl_3$.	14
S24.	gHSQC spectrum of compound **3** in $CDCl_3$.	14

S25.	^1H–^1H gCOSY spectrum of compound **3** in $CDCl_3$.	15
S26.	gHMBC spectrum of compound **3** in $CDCl_3$.	15
S27.	NOESY spectrum of compound **3** in $CDCl_3$.	16
S28.	HR-ESI-MS spectrum of compound **4**.	16
S29.	IR spectrum of compound **4**.	17
S30.	^1H NMR spectrum (400 MHz) of compound **4** in $CDCl_3$.	17
S31.	^{13}C NMR spectrum (100 MHz) of compound **4** in $CDCl_3$.	18
S32.	DEPT spectrum of compound **4** in $CDCl_3$.	18
S33.	gHSQC spectrum of compound **4** in $CDCl_3$.	19
S34.	^1H–^1H gCOSY spectrum of compound **4** in $CDCl_3$.	19
S35.	gHMBC spectrum of compound **4** in $CDCl_3$.	20
S36.	NOESY spectrum of compound **4** in $CDCl_3$.	20

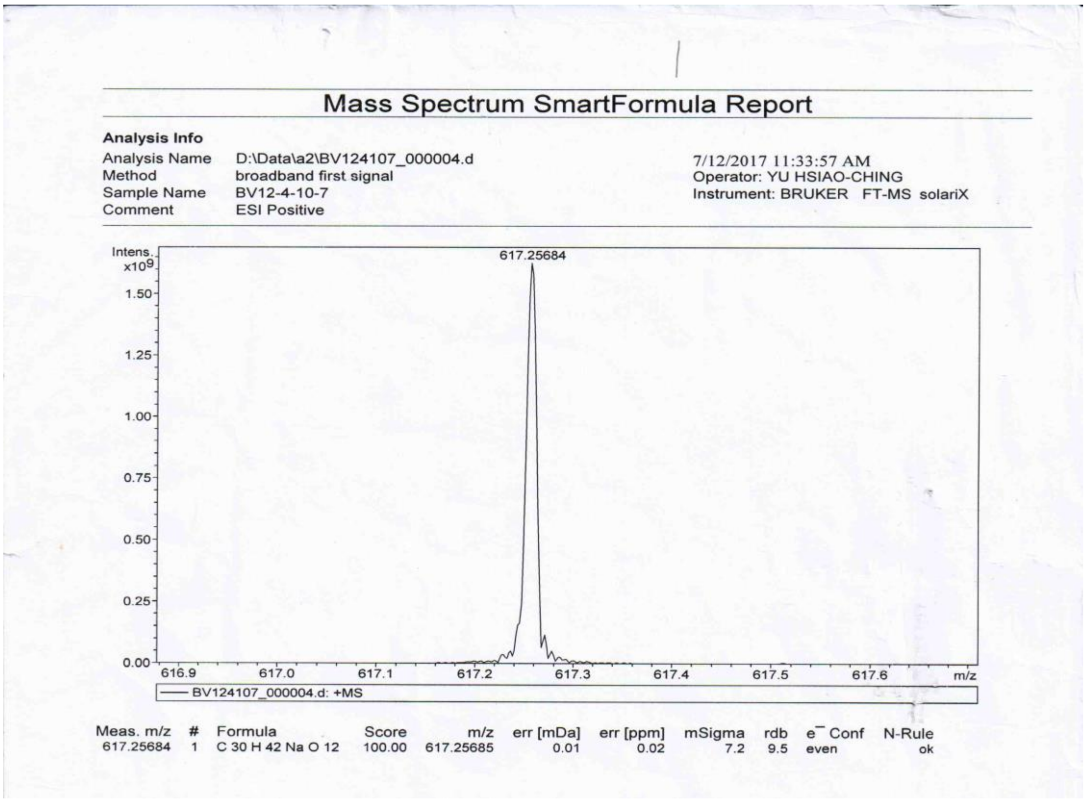

S1. HR-ESI-MS spectrum of compound **1**.

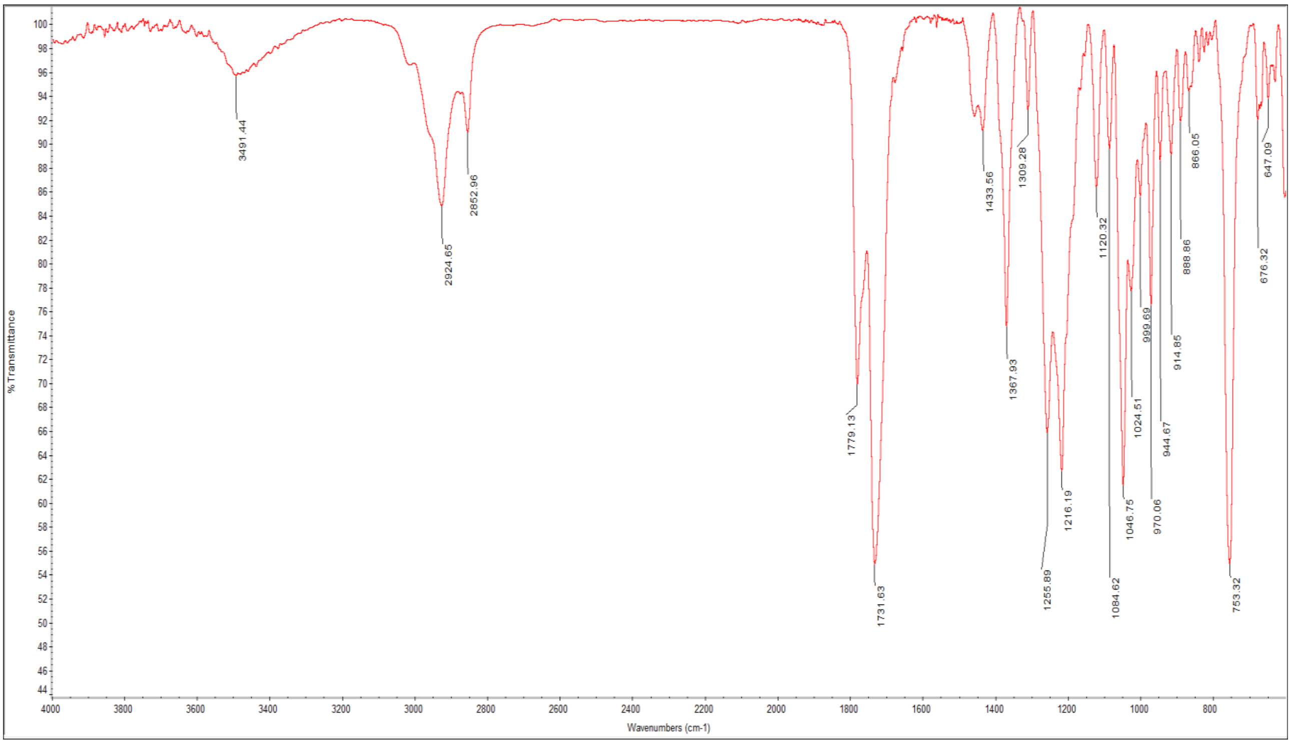

S2. IR spectrum of compound **1**.

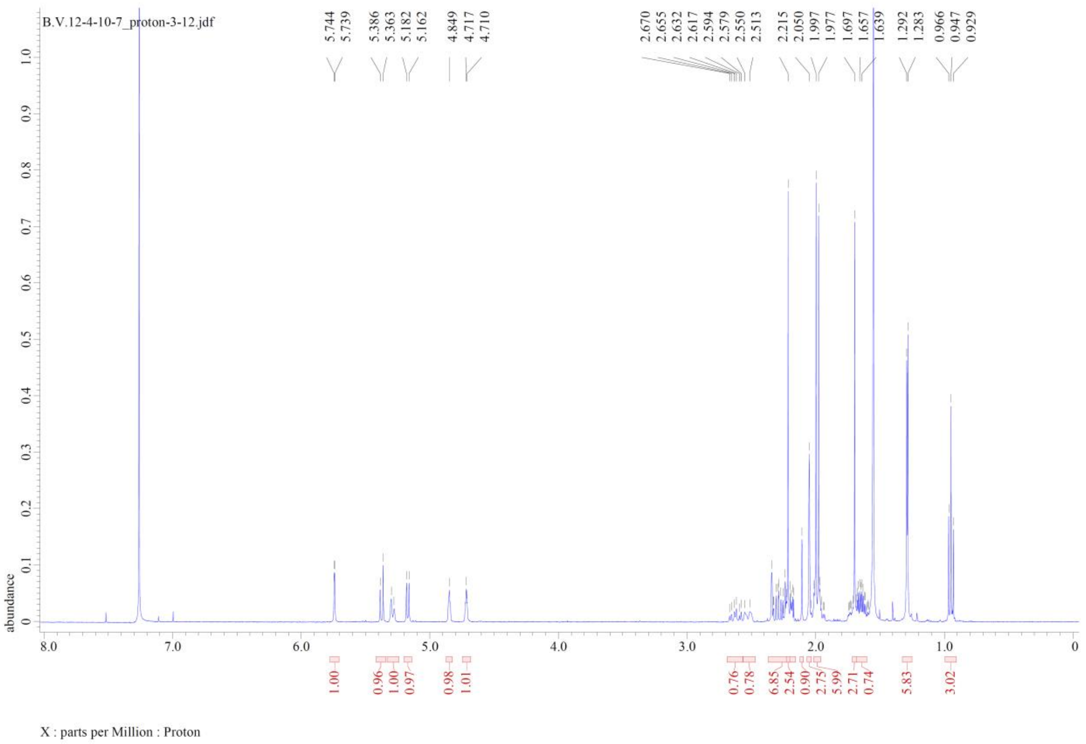

S3. ^{1}H NMR spectrum (400 MHz) of compound **1** in $CDCl_3$

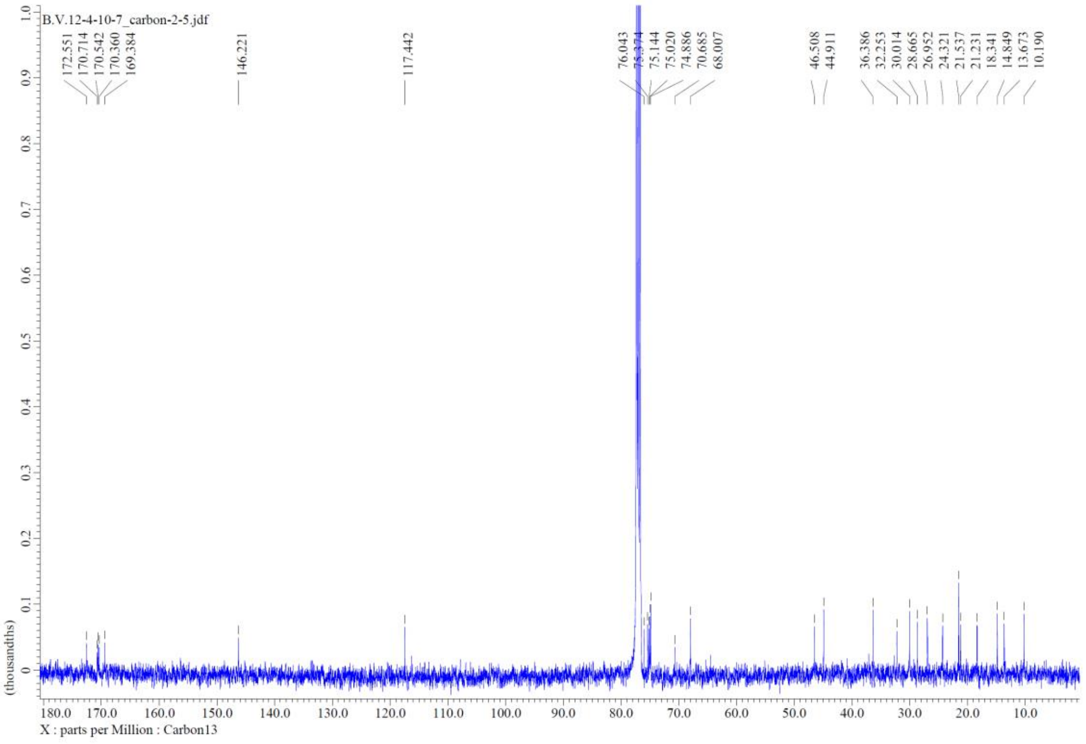

.

S4. 13C NMR spectrum (100 MHz) of compound 1 in CDCl3.

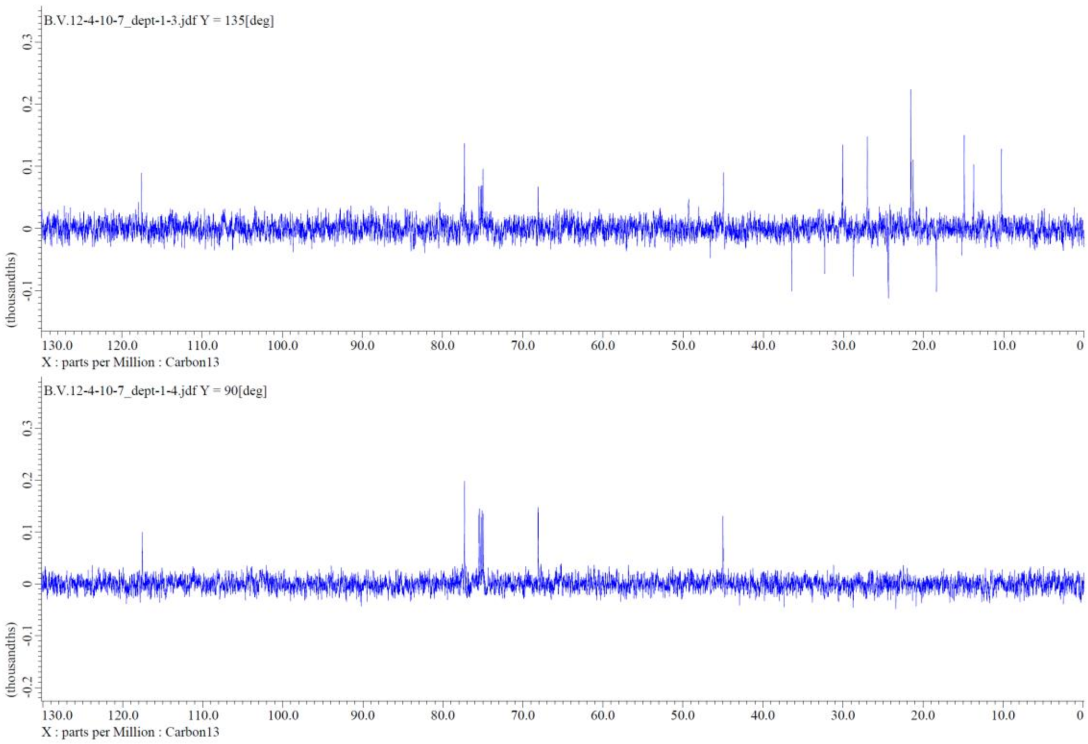

S5. DEPT spectrum of compound **1** in $CDCl_3$.

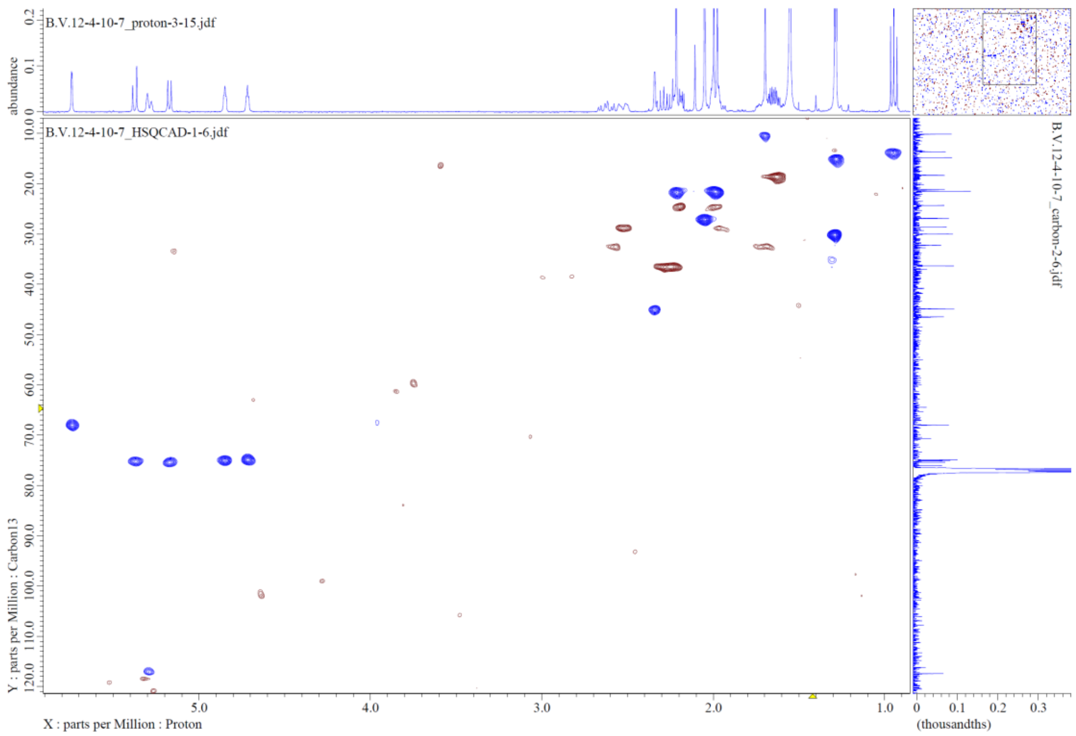

S6. gHSQC spectrum of compound **1** in $CDCl_3$.

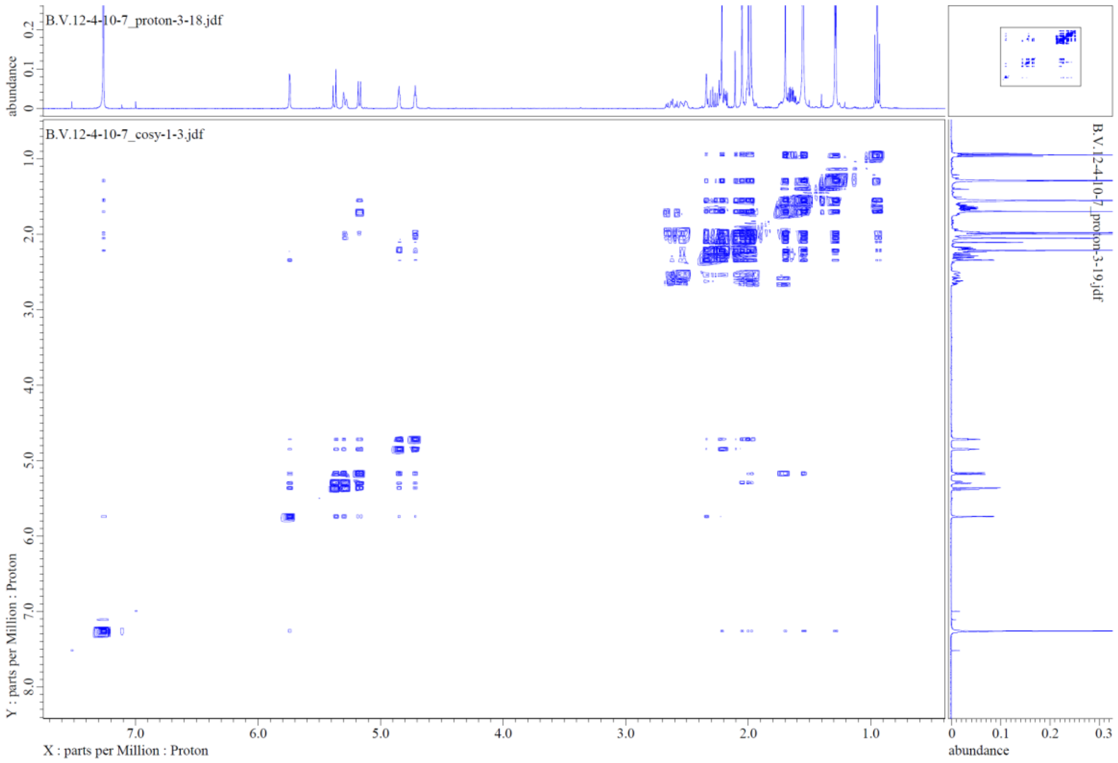

S7. ^{1}H–^{1}H gCOSY spectrum of compound **1** in $CDCl_3$.

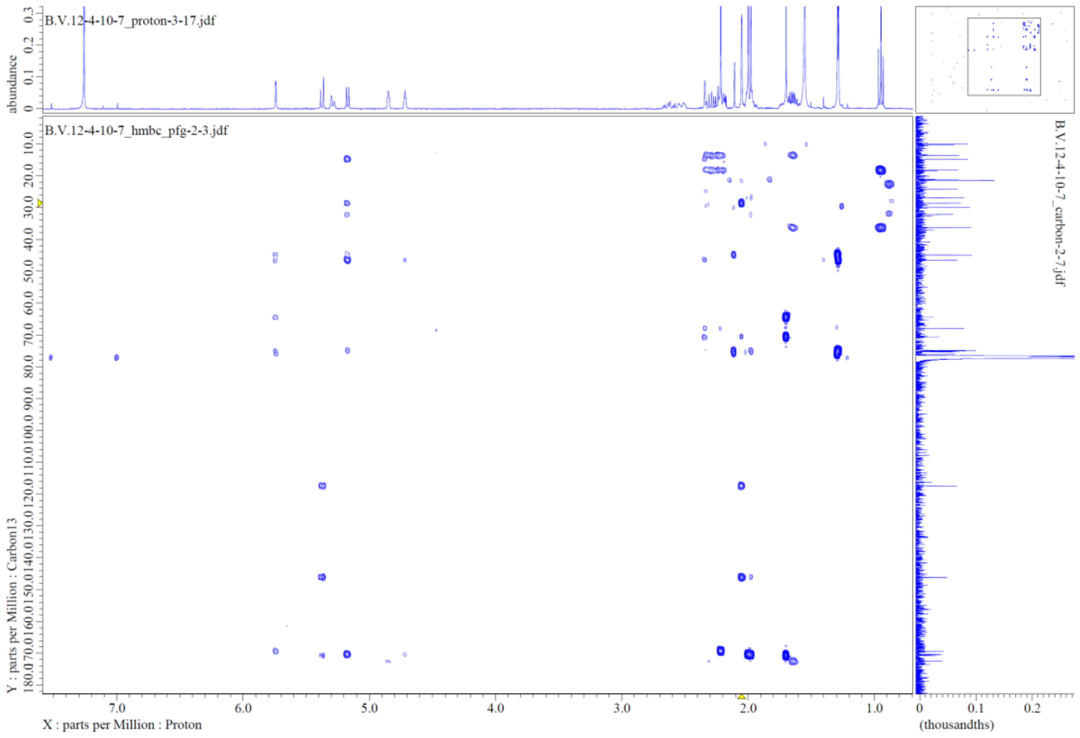

S8. gHMBC spectrum of compound **1** in $CDCl_3$.

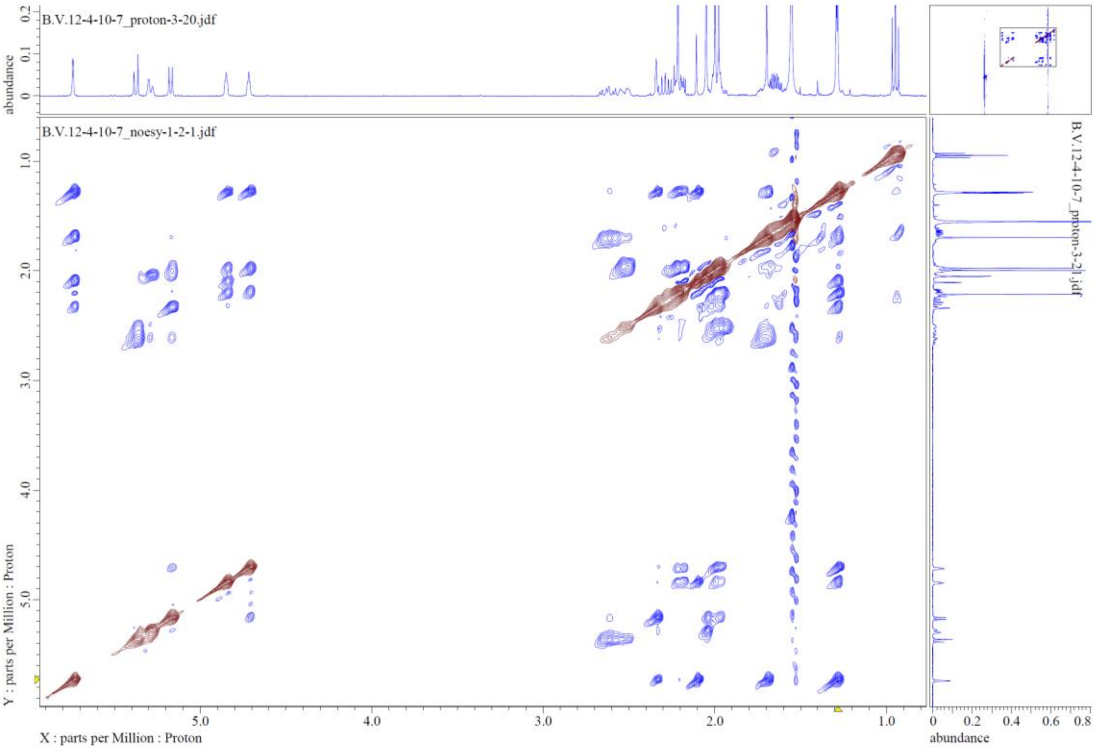

S9. NOESY spectrum of compound **1** in $CDCl_3$.

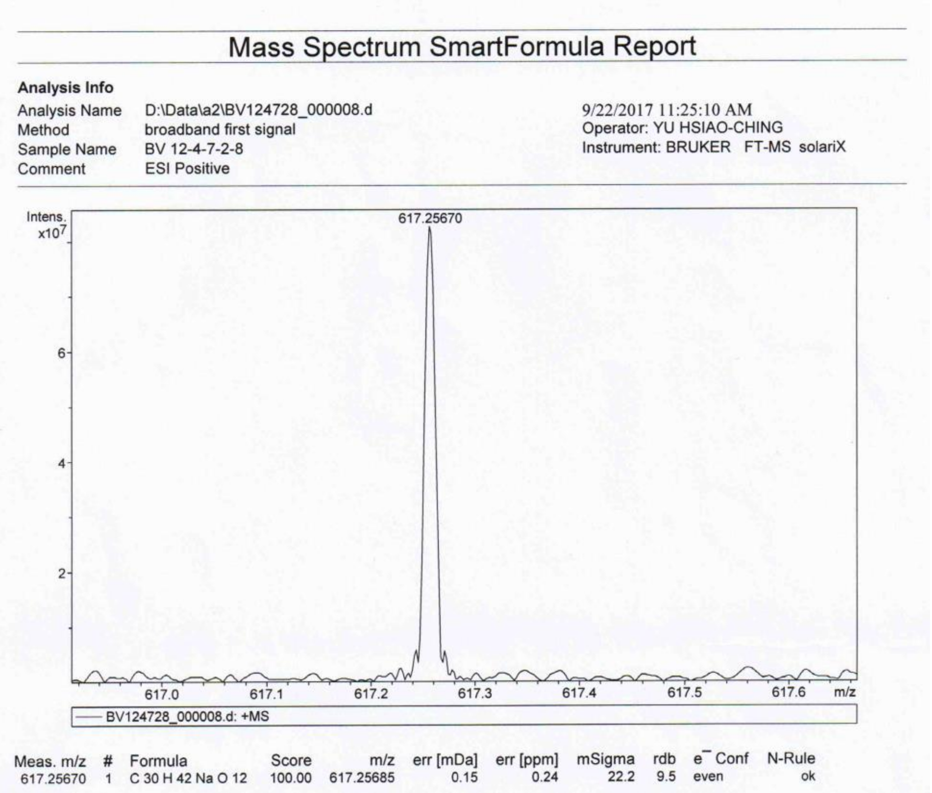

S10. HR-ESI-MS spectrum of compound **2**.

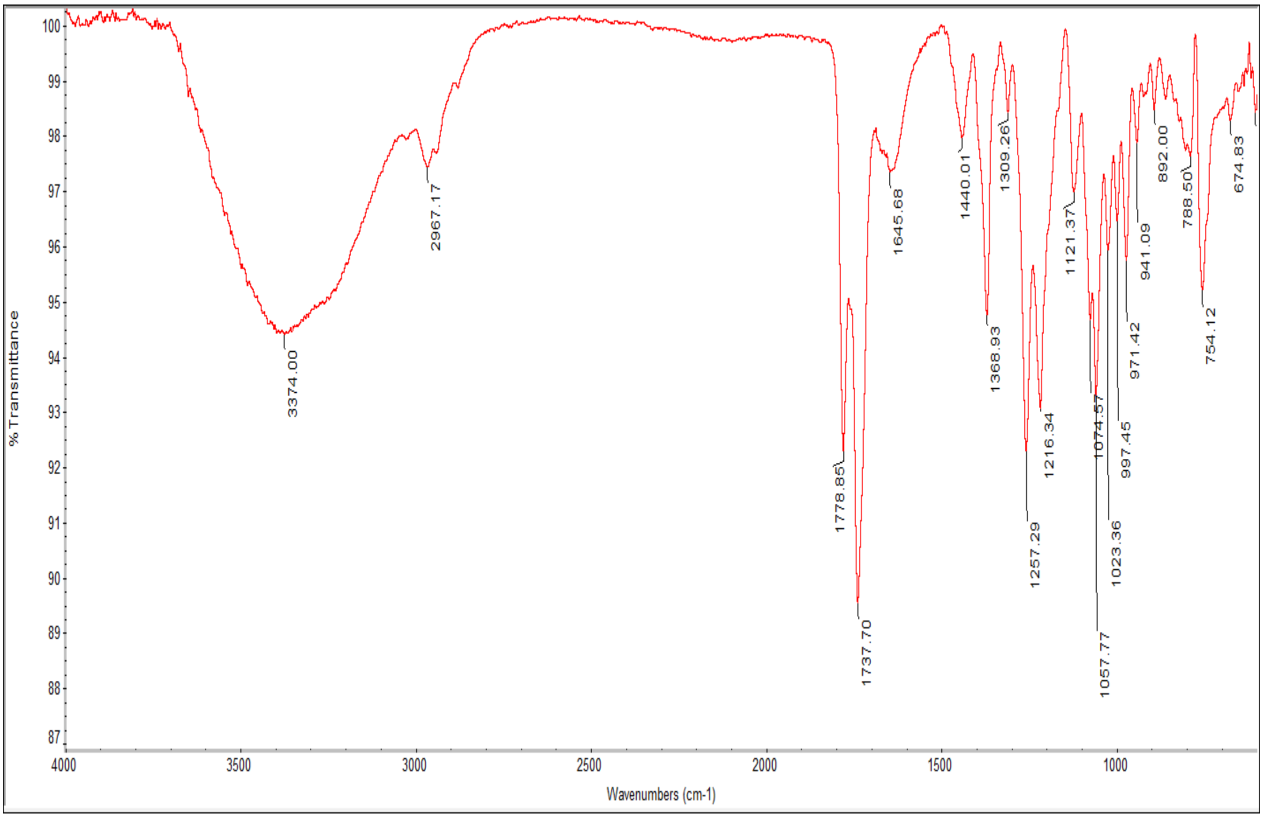

S11. IR spectrum of compound **2**.

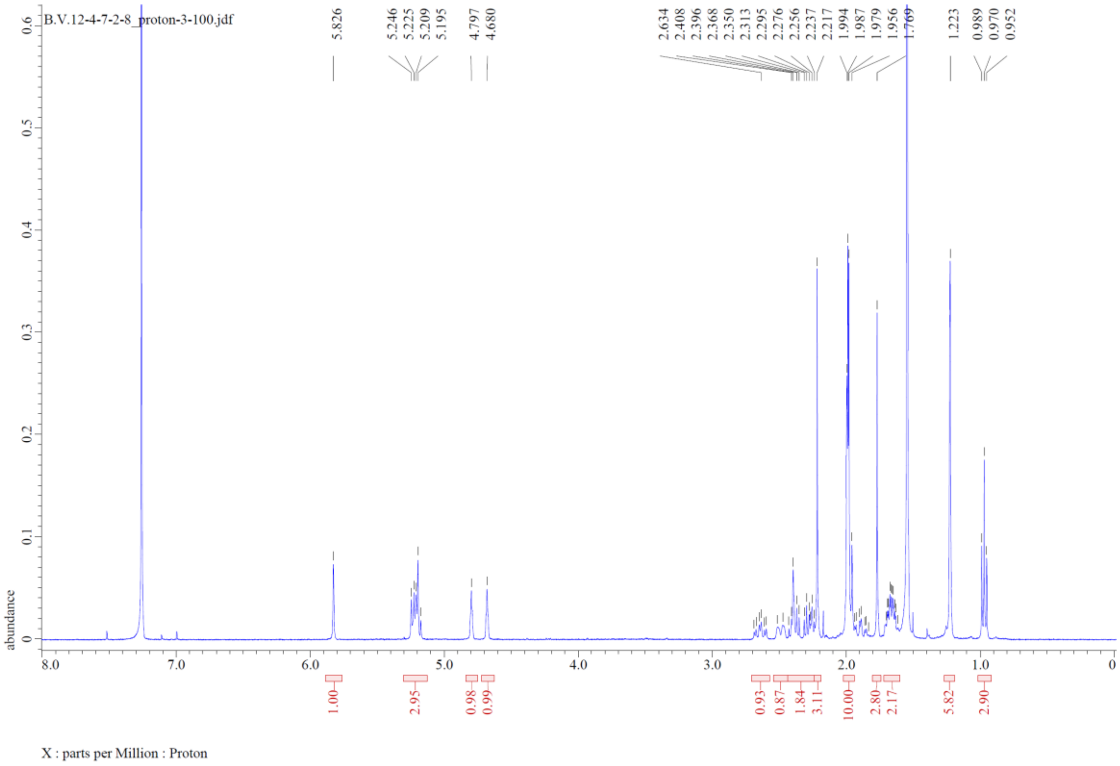

S12. ^{1}H NMR spectrum (400 MHz) of compound **2** in $CDCl_3$.

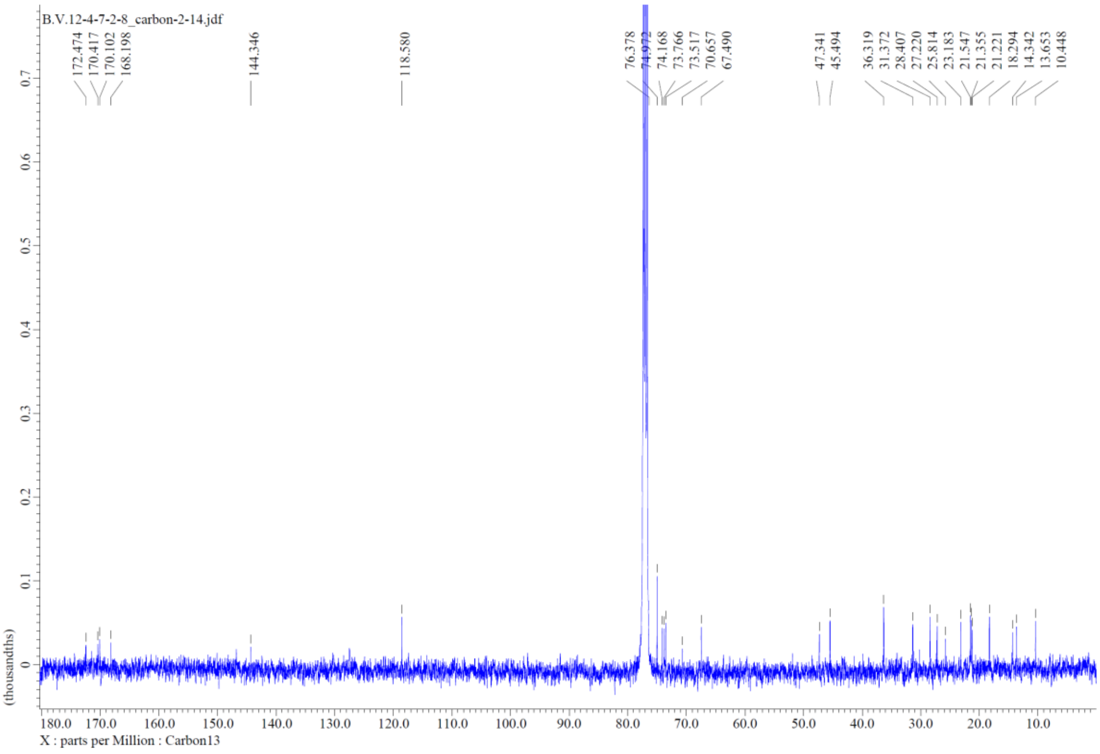

S13. ^{13}C NMR spectrum (100 MHz) of compound **2** in $CDCl_3$.

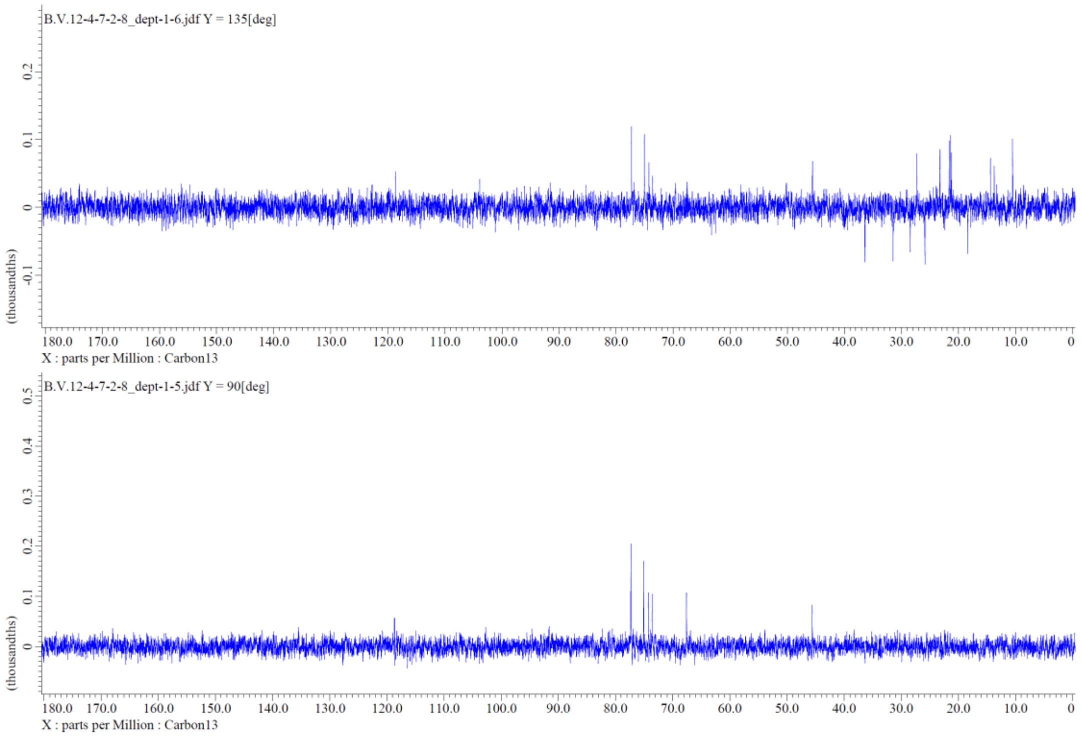

S14. DEPT spectrum of compound **2** in $CDCl_3$.

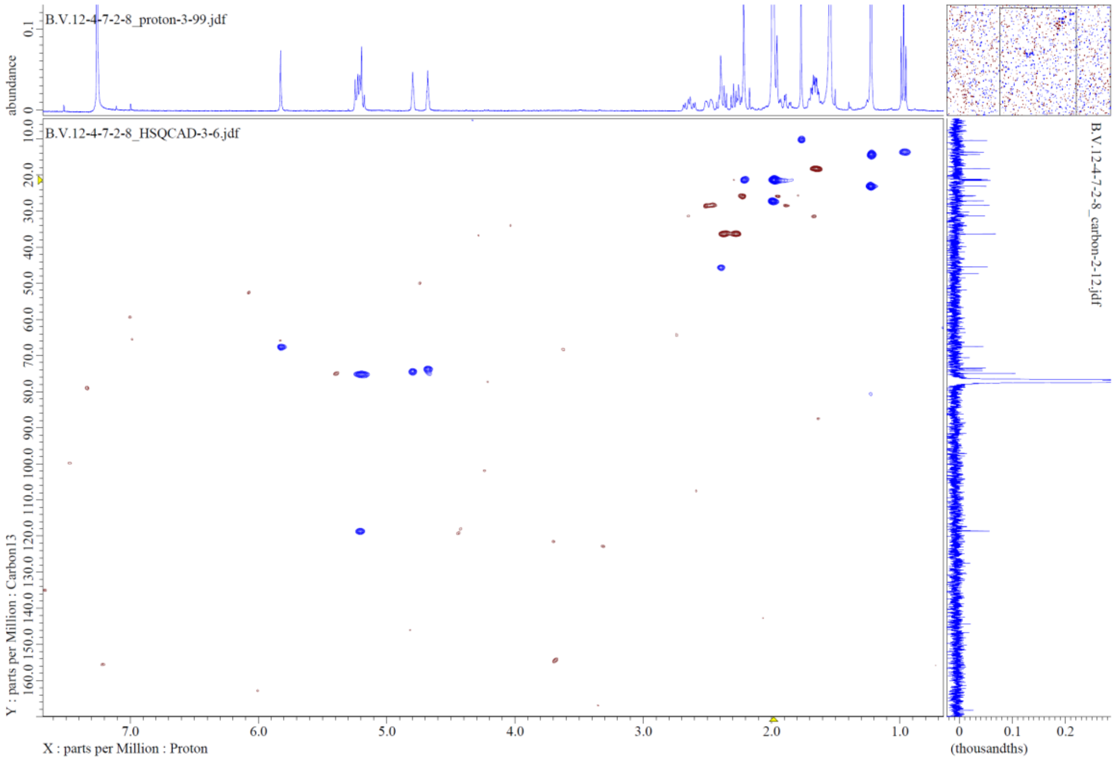

S15. gHSQC spectrum of compound **2** in $CDCl_3$.

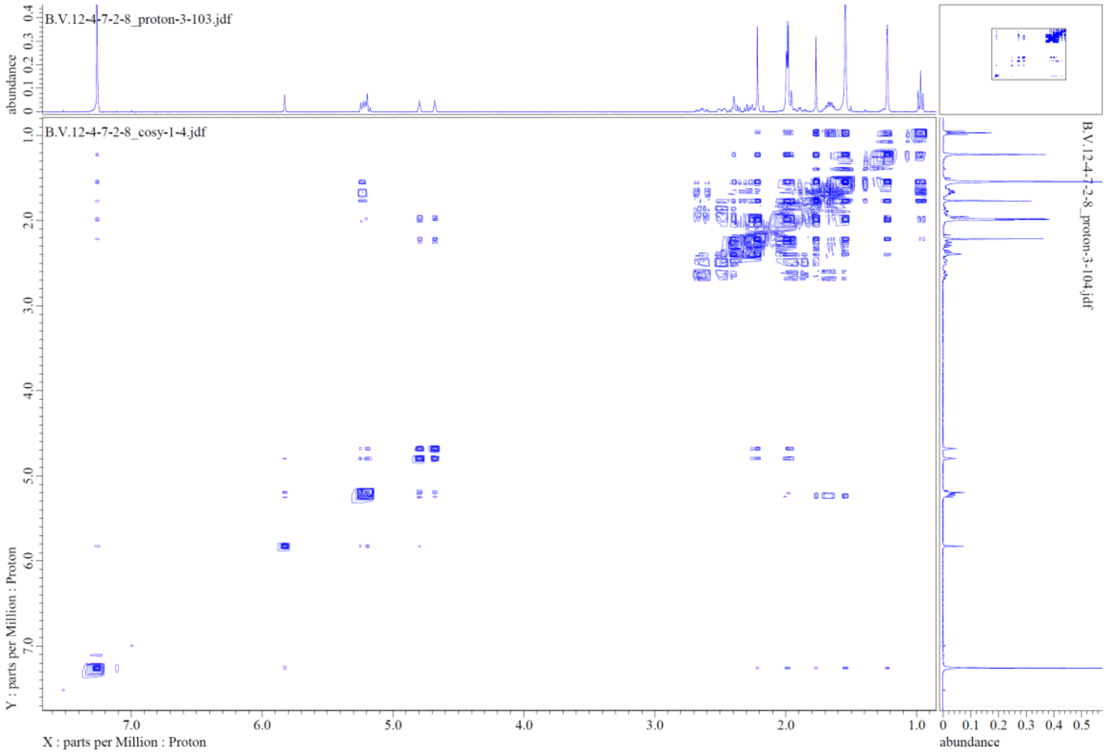

S16. ^{1}H–^{1}H gCOSY spectrum of compound **2** in $CDCl_3$.

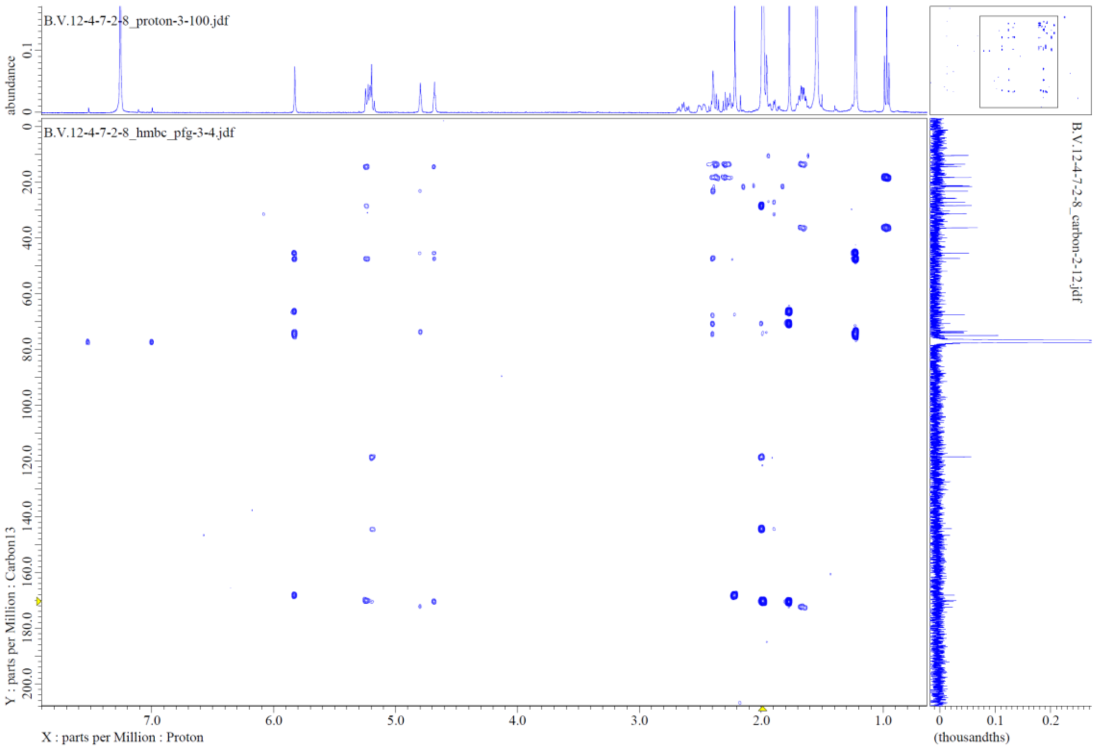

S17. gHMBC spectrum of compound **2** in $CDCl_3$.

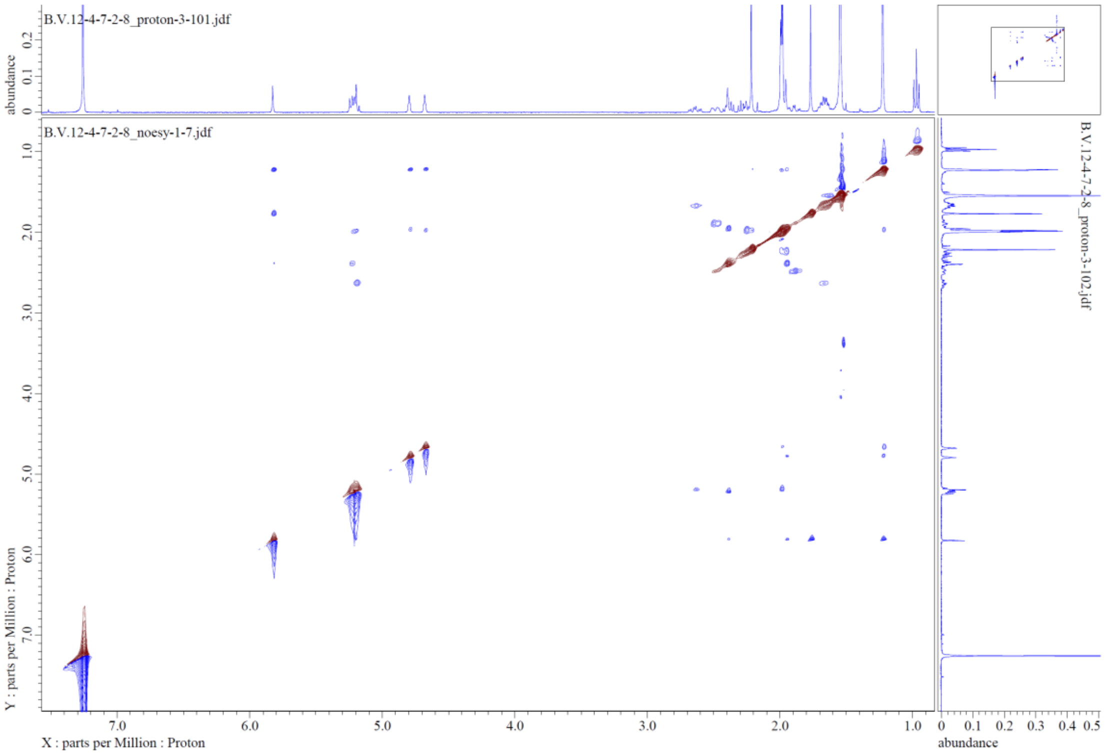

S18. NOESY spectrum of compound **2** in $CDCl_3$.

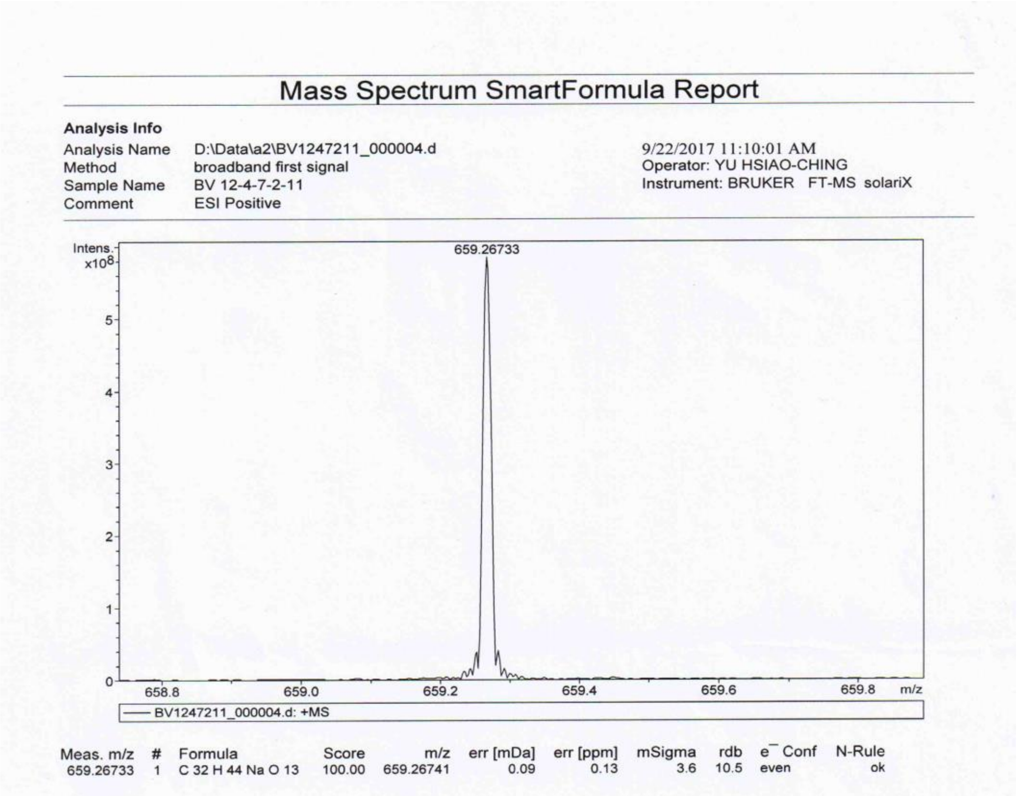

S19. HR-ESI-MS spectrum of compound **3**.

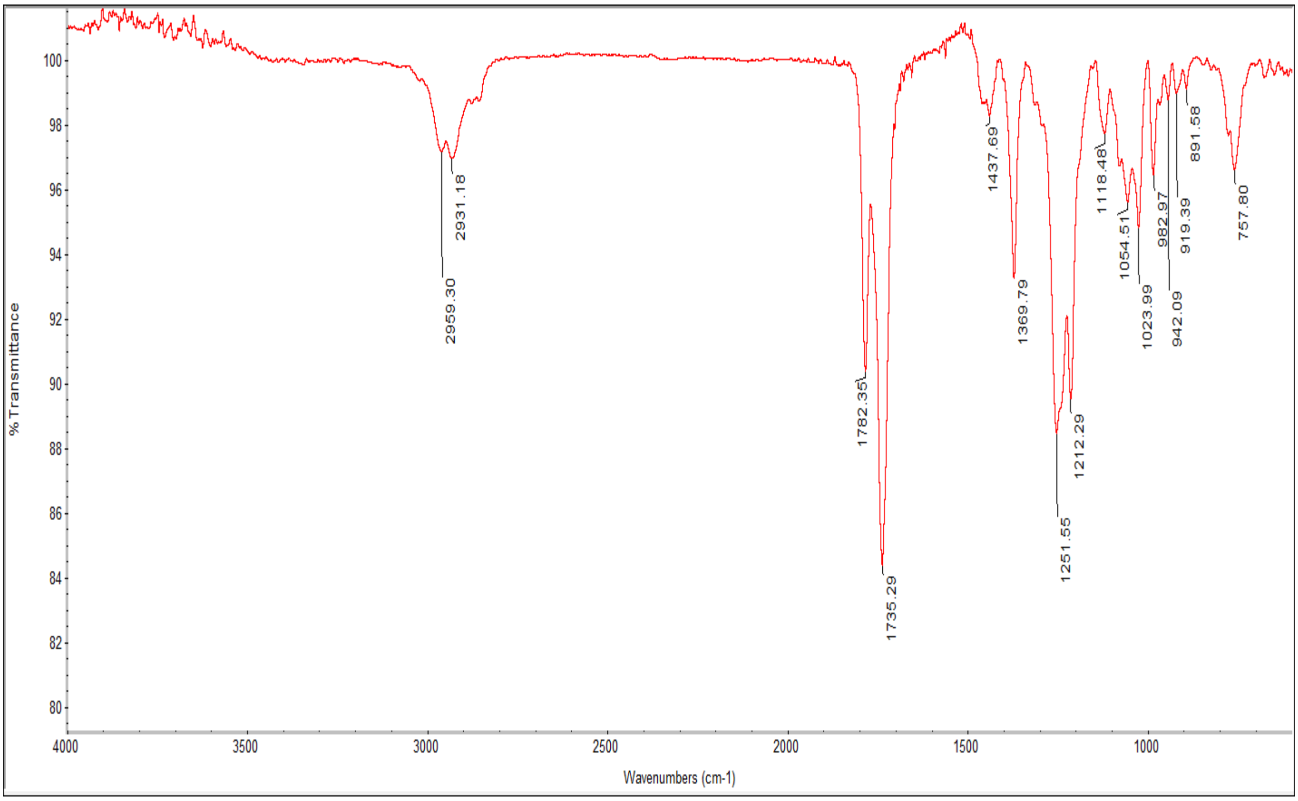

S20. IR spectrum of compound **3**.

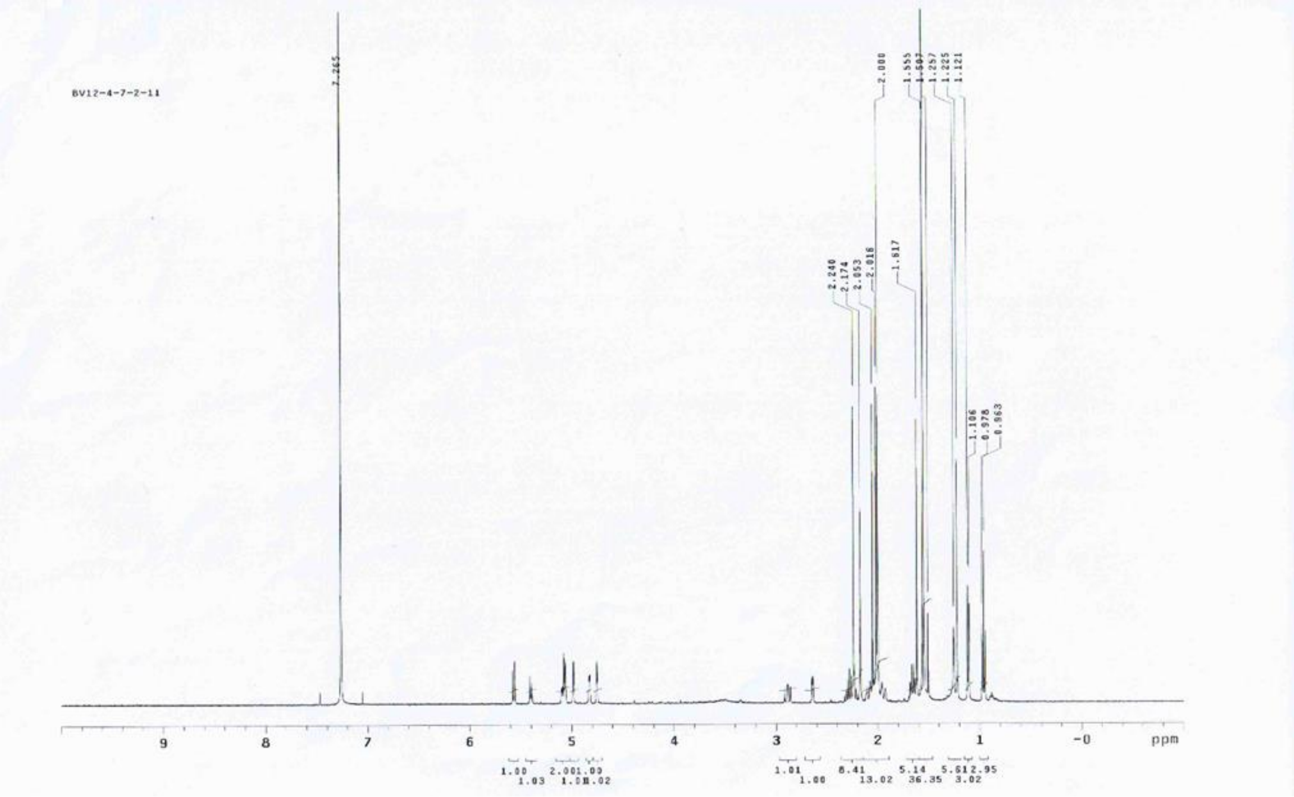

S21. ^{1}H NMR spectrum (500 MHz) of compound **3** in $CDCl_3$.

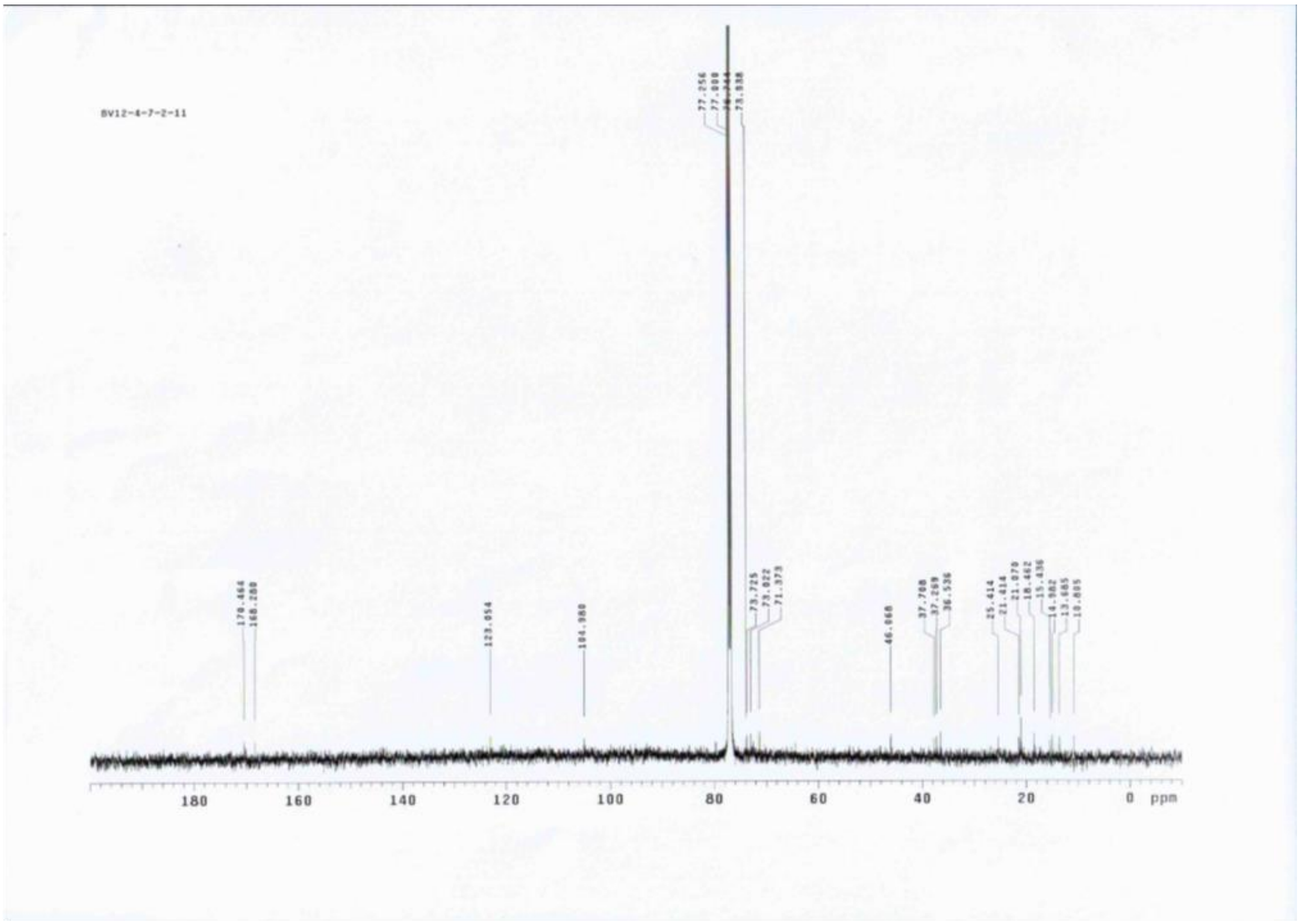

S22. ^{13}C NMR spectrum (125 MHz) of compound **3** in $CDCl_3$.

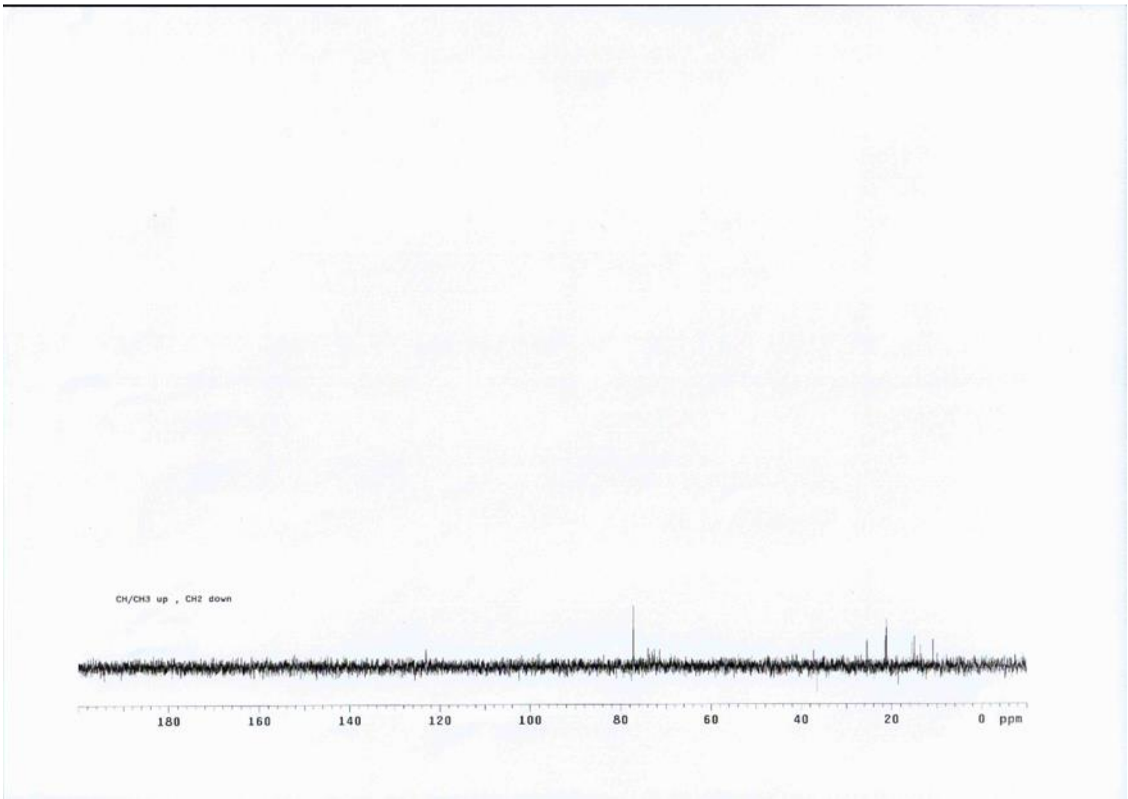

S23. DEPT spectrum (500 MHz) of compound **3** in $CDCl_3$.

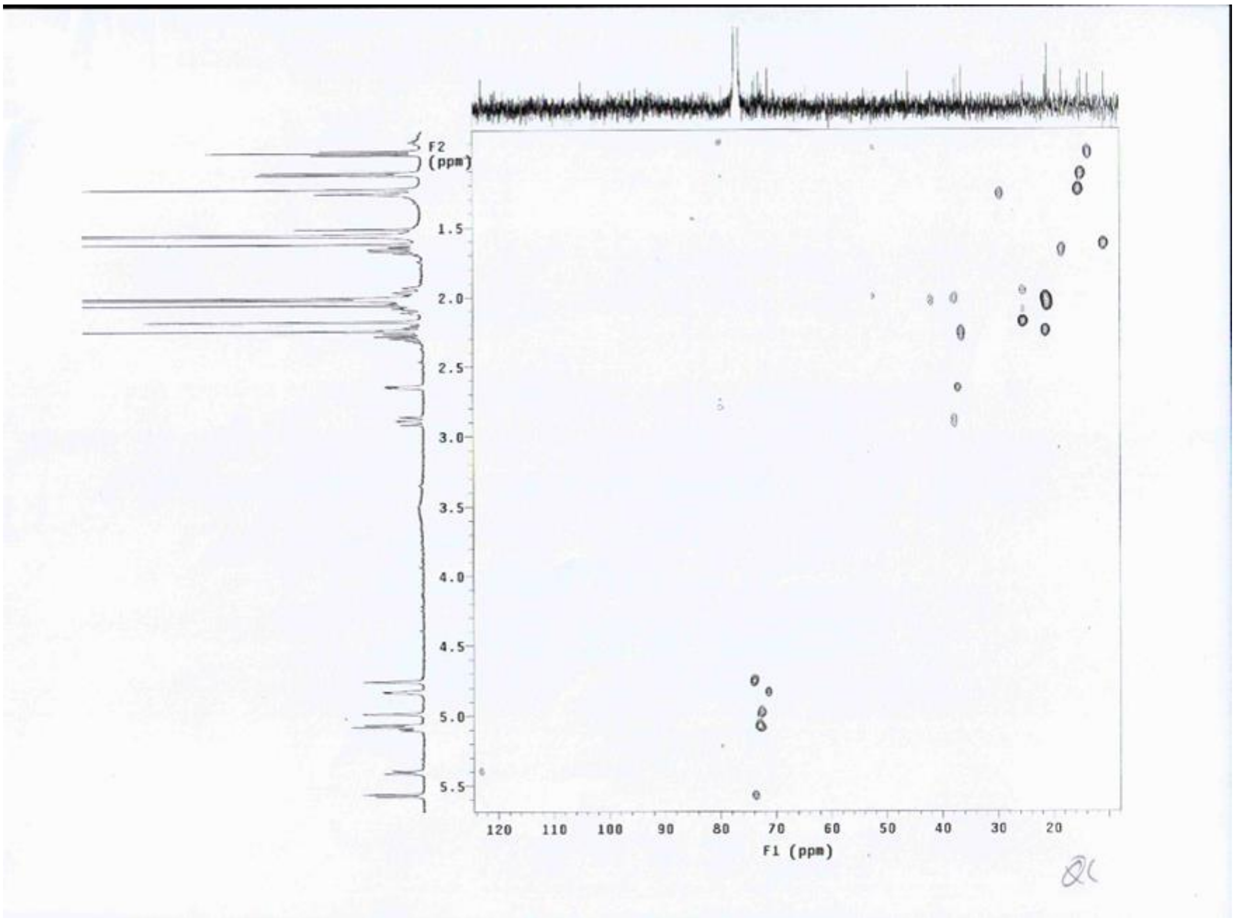

S24. gHSQC spectrum of compound **3** in $CDCl_3$.

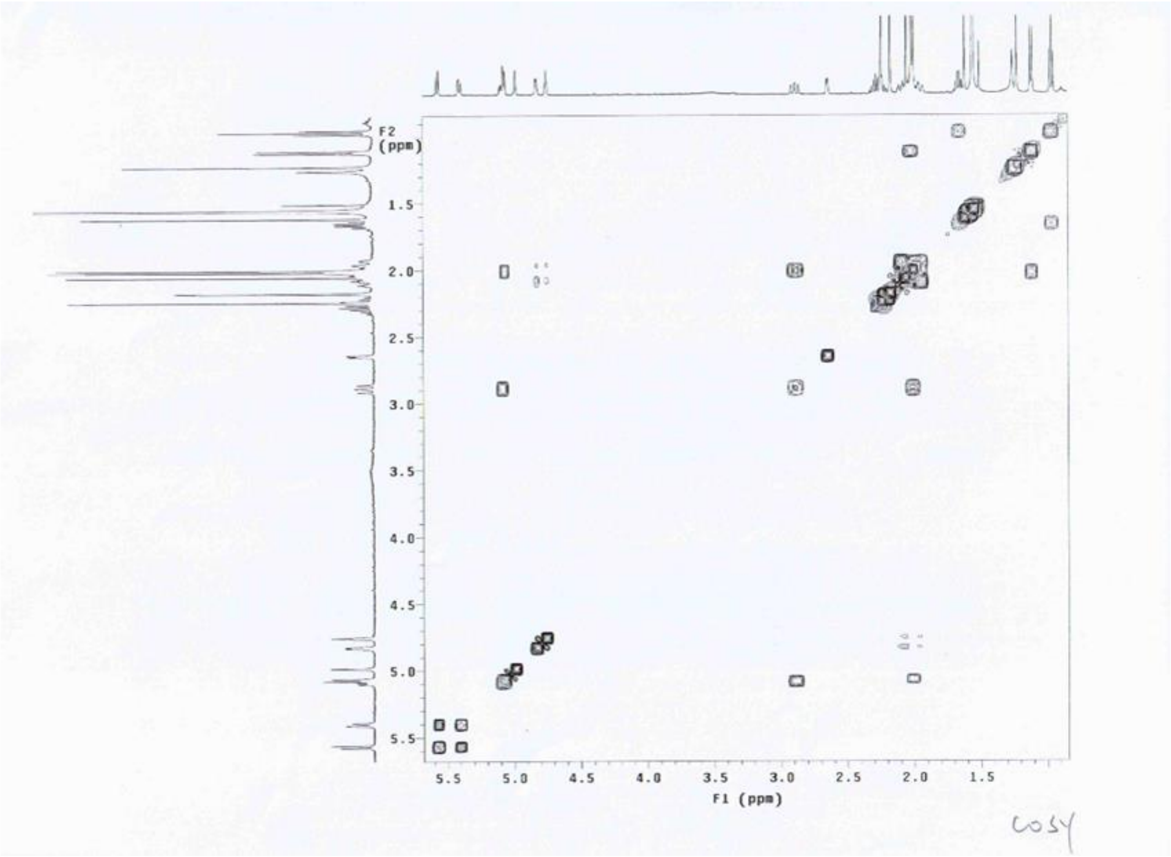

S25. ^{1}H–^{1}H gCOSY spectrum of compound **3** in $CDCl_3$.

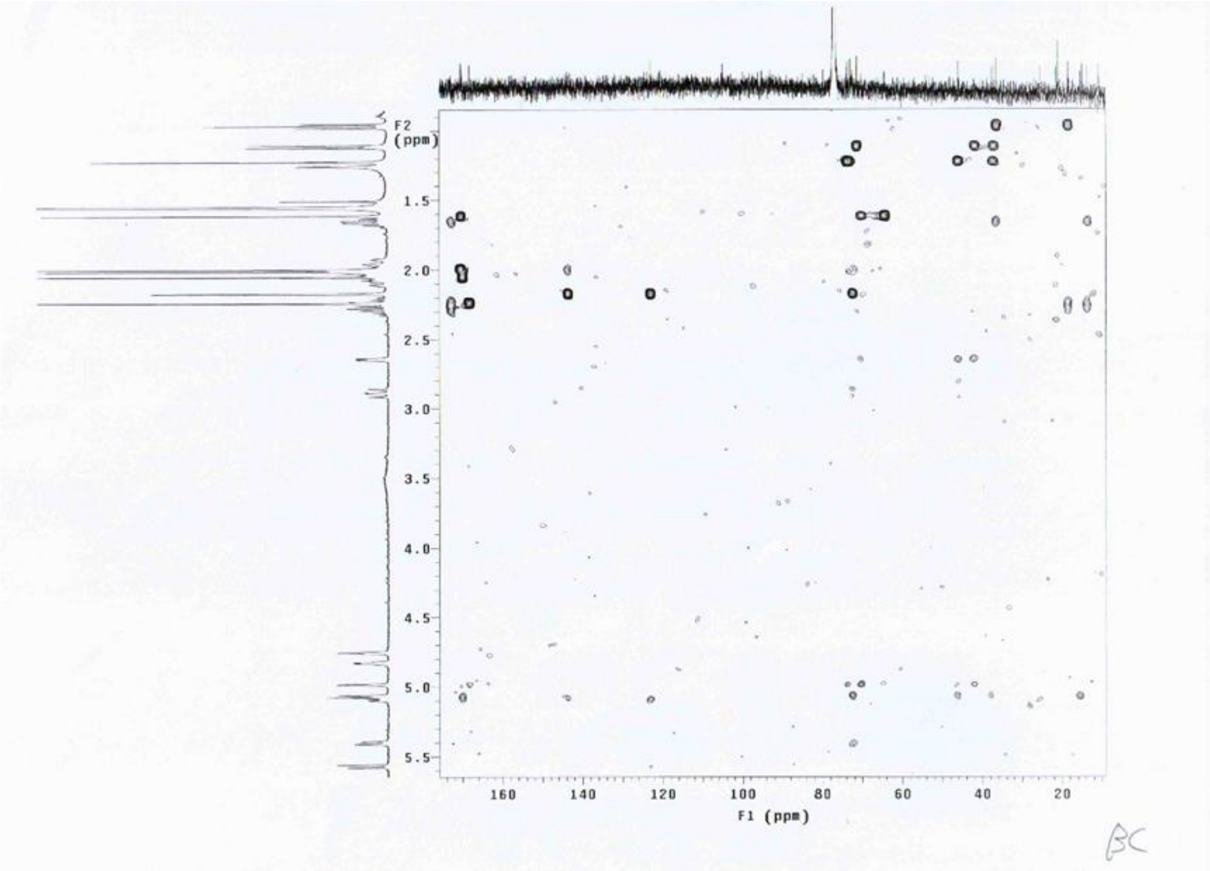

S26. gHMBC spectrum of compound **3** in $CDCl_3$.

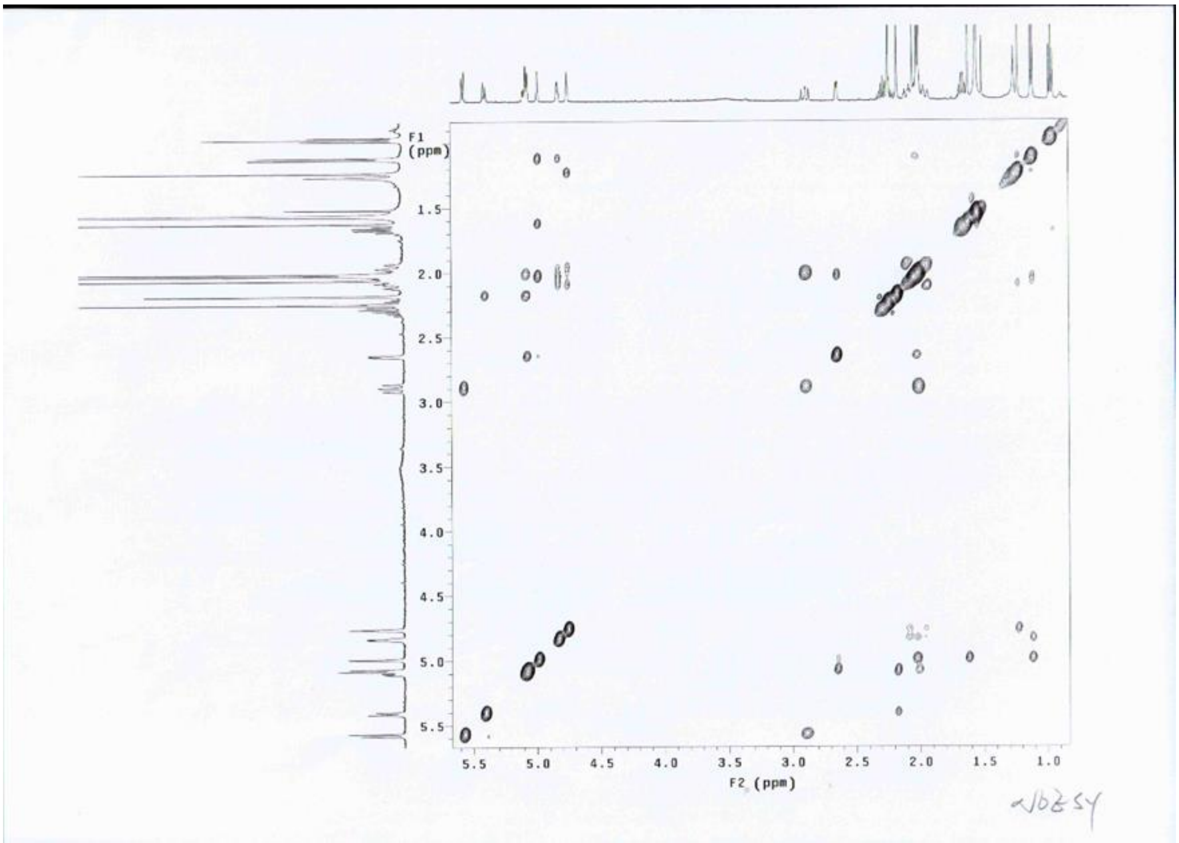

S27. NOESY spectrum of compound **3** in $CDCl_3$.

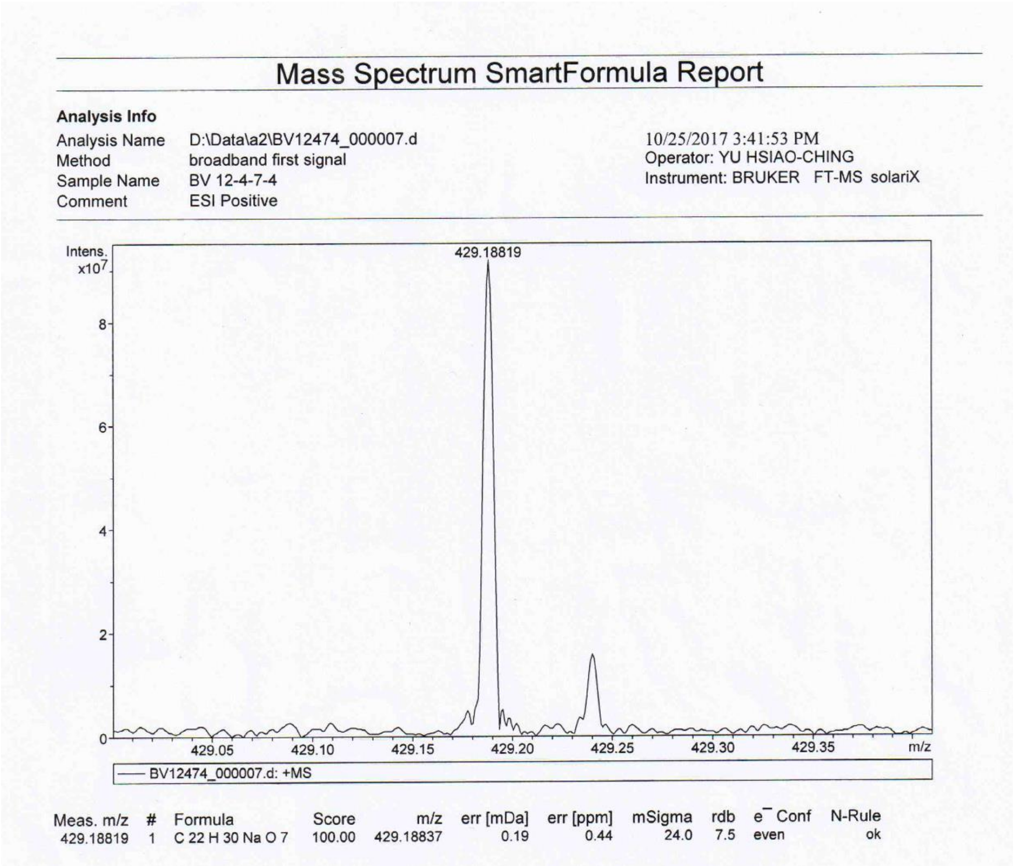

S28. HR-ESI-MS spectrum of compound 4.

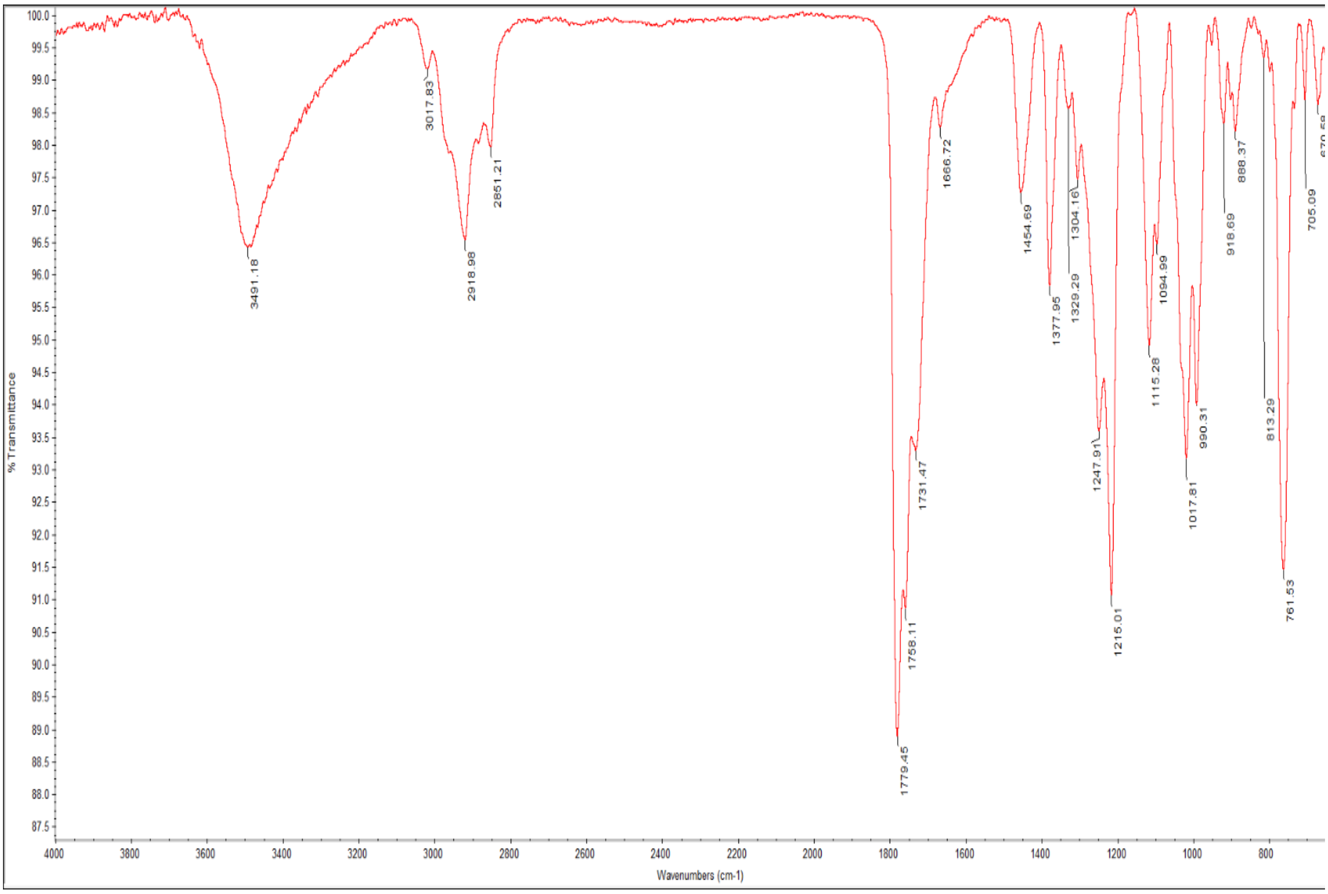

S29. IR spectrum of compound **4**.

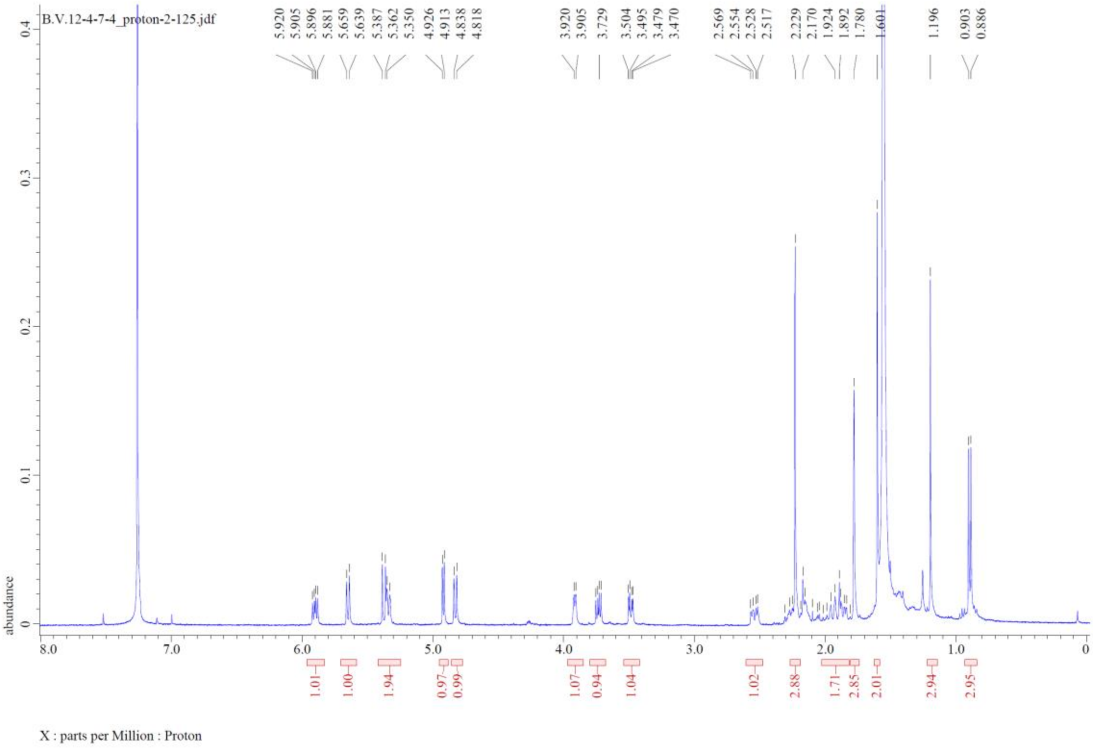

S30. ^{1}H NMR spectrum (400 MHz) of compound **4** in $CDCl_3$.

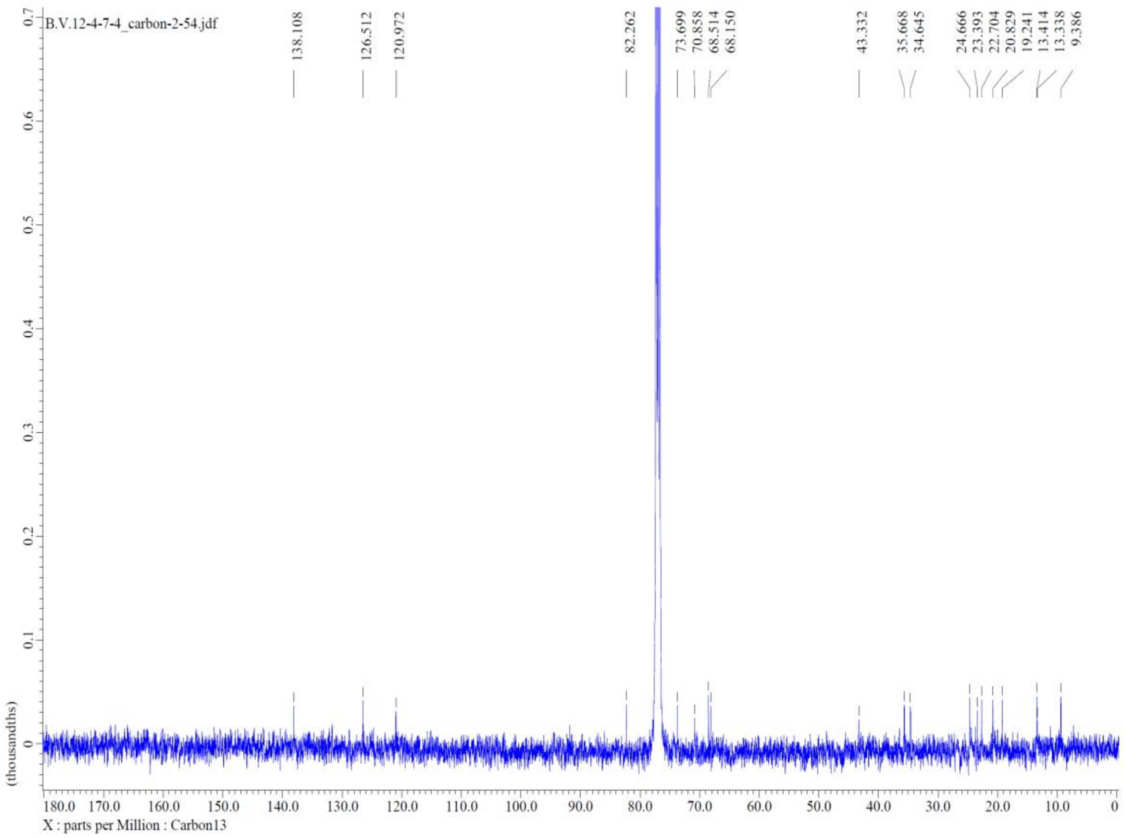

S31. ^{13}C NMR spectrum (400 MHz) of compound **4** in $CDCl_3$.

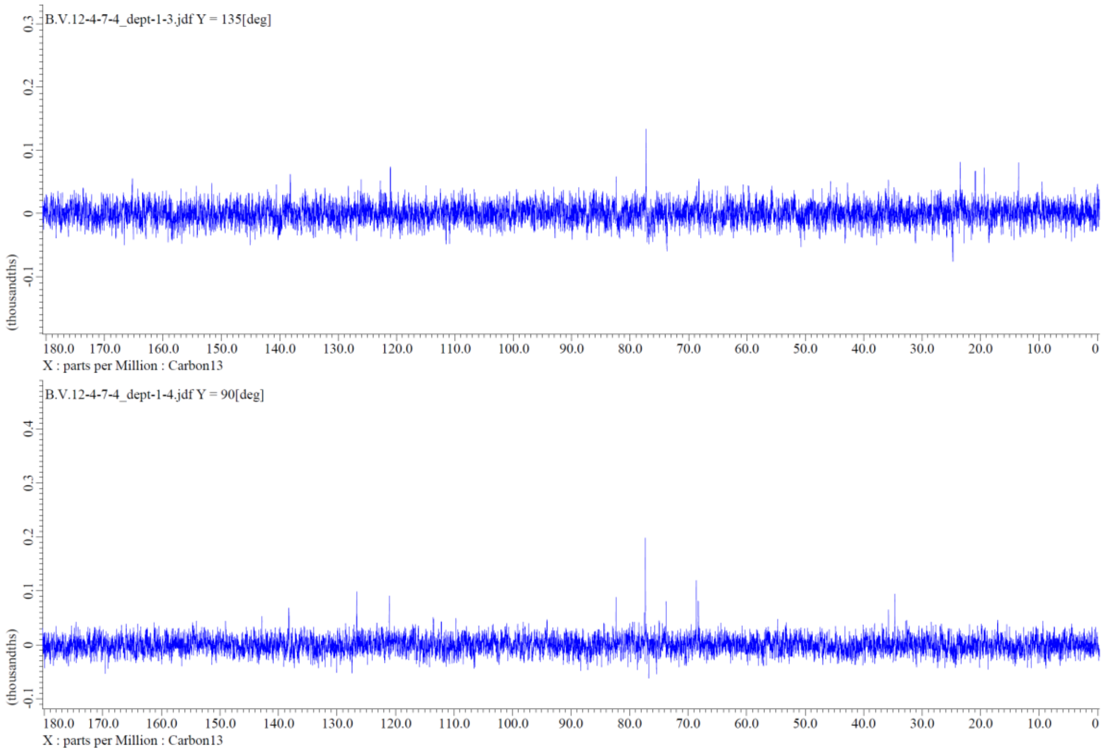

S32. DEPT spectrum (400 MHz) of compound **4** in $CDCl_3$.

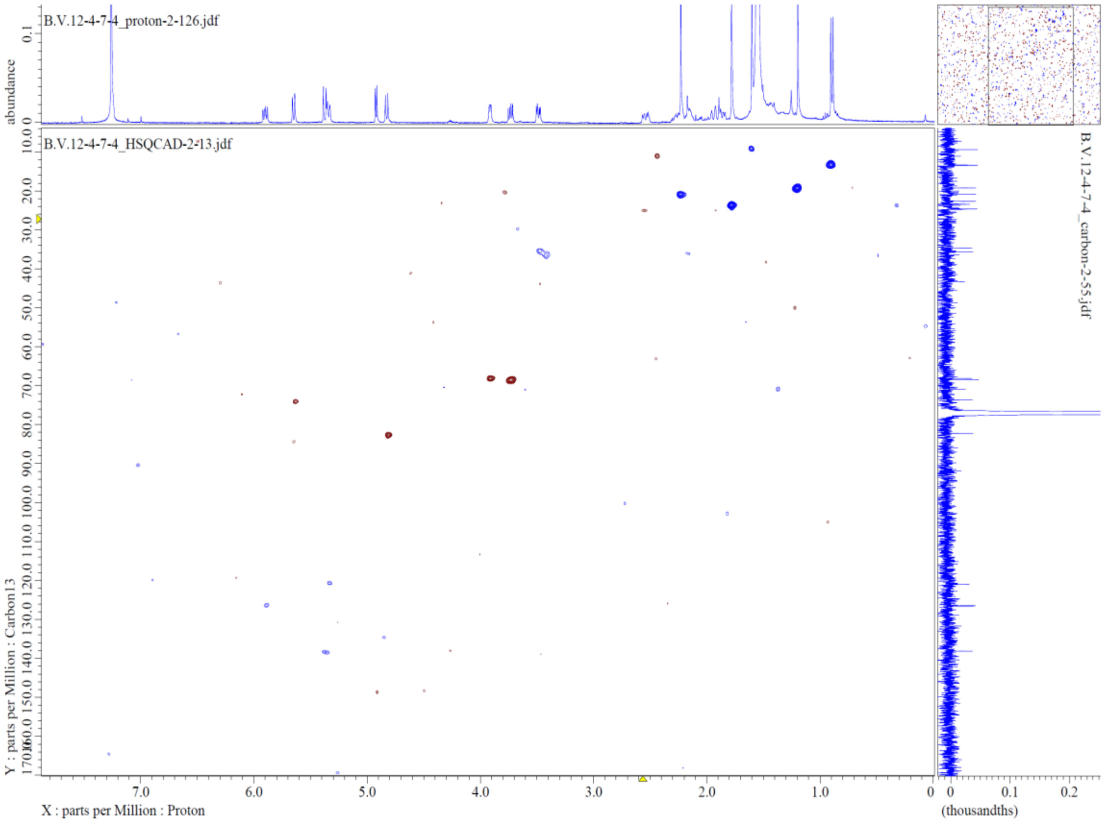

S33. gHSQC spectrum of compound **4** in $CDCl_3$.

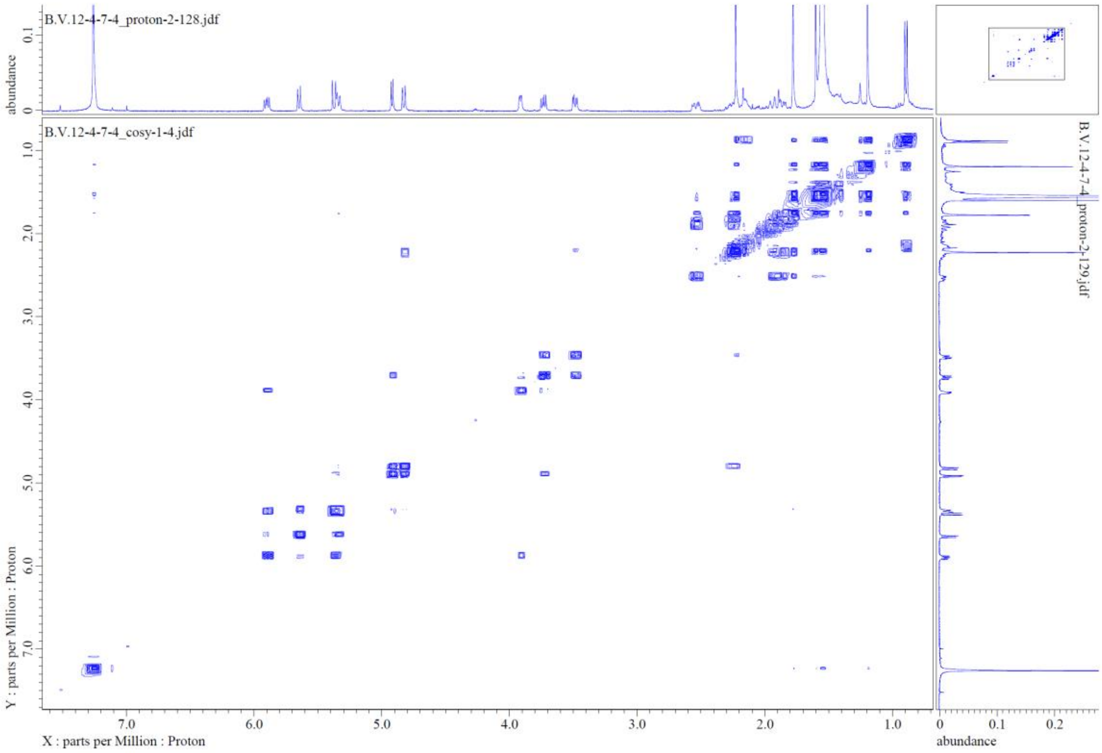

S34. ^{1}H–^{1}H gCOSY spectrum of compound **4** in $CDCl_3$.

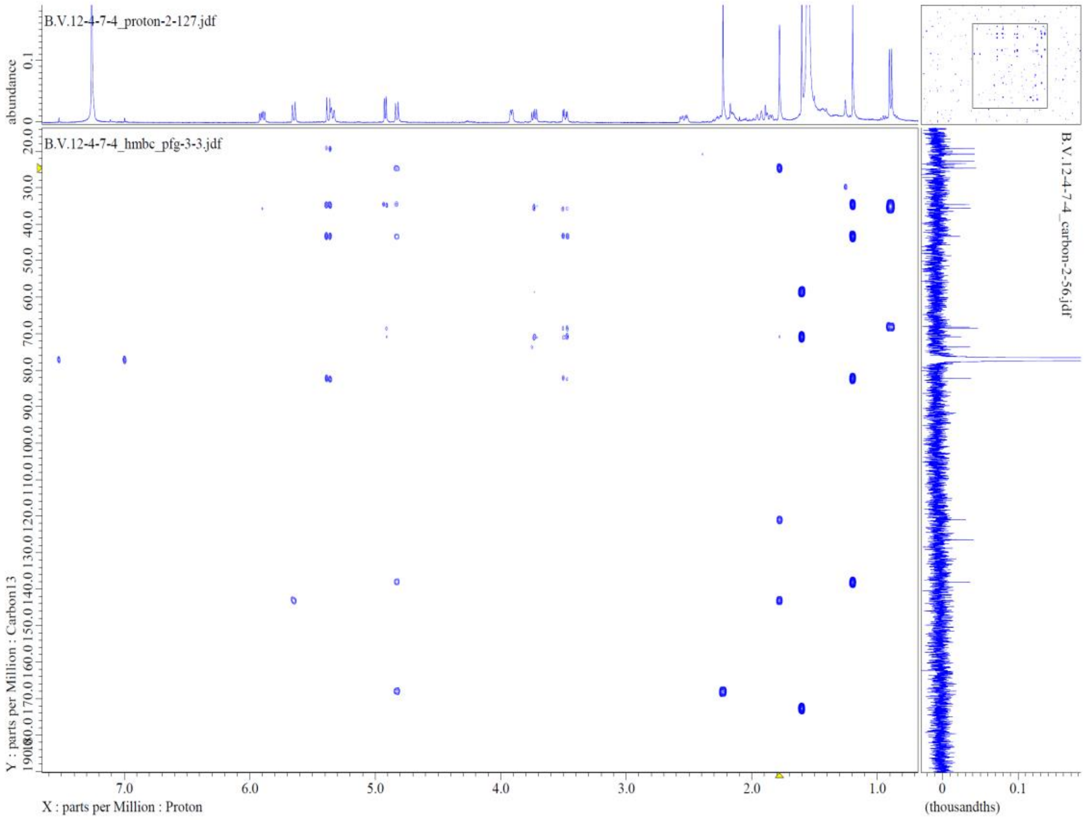

S35. gHMBC spectrum of compound **4** in $CDCl_3$.

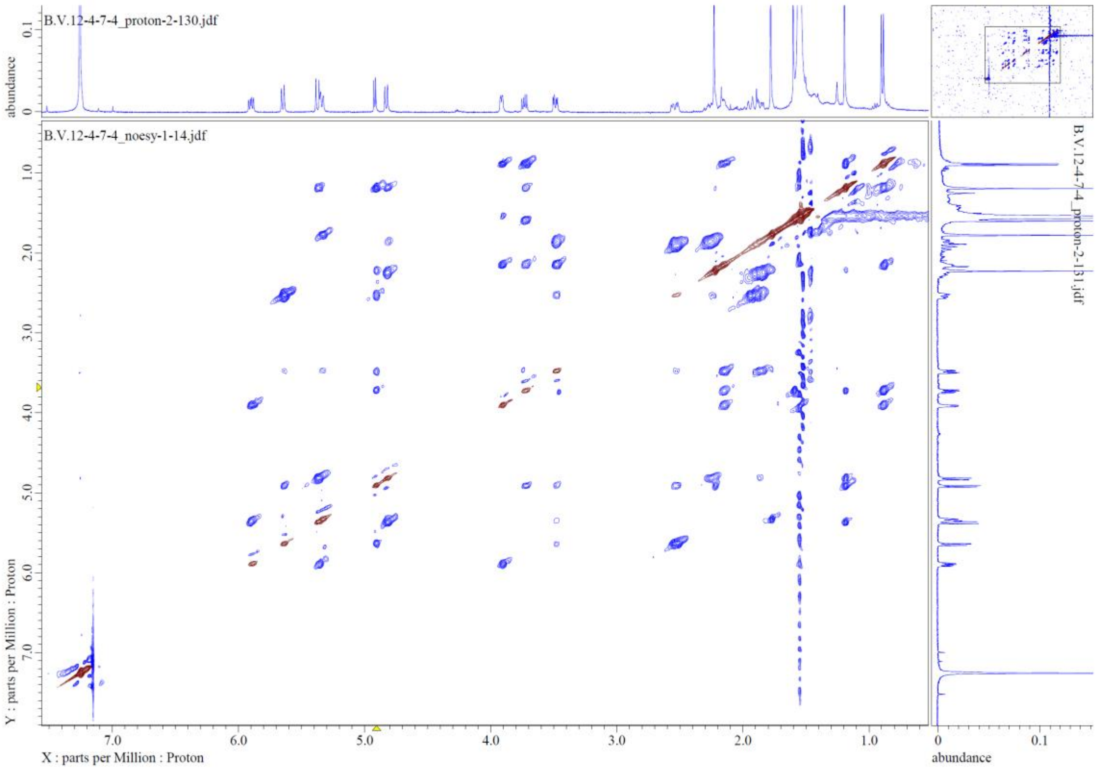

S36. NOESY spectrum of compound **4** in $CDCl_3$.
